# Revisiting the “Cluster‐In‐Solvent” Approach for Computational Spectroscopy: The Vibrational Circular Dichroism as a Test Case

**DOI:** 10.1002/jcc.70144

**Published:** 2025-06-20

**Authors:** Srilatha Arra, Isabella Daidone, Massimiliano Aschi

**Affiliations:** ^1^ Department of Physical and Chemical Sciences University of L'Aquila L'Aquila Italy

**Keywords:** cluster analysis, computational spectroscopy, essential dynamics, molecular dynamics, vibrational circular dichroism

## Abstract

The cluster‐in‐solvent approach, that is, the use of the quantum‐mechanical calculation of a spectral observable on a significant number of solute–solvent clusters extracted from semi‐classical simulations, is widely used in computational spectroscopy. However, identifying relevant coordinates for cluster selection remains a challenge. We previously developed the Ellipsoid Method for Cluster‐in‐Solvent (EMCS), an automated strategy for unbiased identification and statistical weighting of clusters. Yet, for larger solutes, EMCS can yield overly large solvent clusters that hinder conformational analysis. Here, we introduce a simple extension of EMCS that reduces cluster size, enabling its application to medium‐to‐large solutes. The method is validated through the computation of Vibrational Circular Dichroism (VCD) spectra, which are highly sensitive to solute–solvent interactions. Test cases include aqueous L‐alanine, aqueous dialanine, and (1S,2S)‐trans‐1‐amino‐2‐indanol in DMSO. For L‐alanine and trans‐1‐amino‐2‐indanol, computed spectra closely match experiment, with root‐mean‐square‐deviation (RMSD) values of 10.3 and 8.0, respectively, consistent with previous benchmarks. For aqueous dialanine, the main spectral features were reproduced, though discrepancies in the fine structure remain, likely due to limitations in capturing subtle solvation effects. Overall, the refined EMCS protocol enables efficient and non‐arbitrary sampling of solute–solvent clusters, offering a valuable tool for the structural analysis of solvation shells in complex molecular systems.

## Graphical abstract

A computational method is proposed to unambiguously identify solute–solvent clusters of different sizes from semi‐classical Molecular Dynamics simulations. The method is tested through the modeling of the Vibrational Circular Dichroism spectral features of two systems taken as test cases.

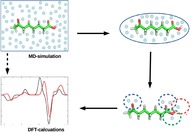

## 1 Introduction

Among the various methods proposed in the context of Soft‐Matter Computational Spectroscopy [1, 2, 3, 4, 5, 6, 7] the “cluster‐in‐solvent” approach has proven to be one of the most extensively used. In this computational scheme [8, 9, 10, 11] the spectral observables are calculated through quantum‐chemical calculations on a series of solute–solvent clusters of limited size, extracted from semi‐classical Molecular Dynamics (MD) or Monte Carlo (MC) simulations. One critical point of this protocol, beyond the obvious issue concerning the quality of the force field, is the criterion followed to choose the solute–solvent cluster internal coordinates necessary for univocally identifying the corresponding conformations. One possible strategy is based on the brute‐force approach in which a huge number of uncorrelated solute–solvent clusters is selected from the MD or MC simulation [12, 13]. Alternatively, the conformational analysis can be addressed following, when possible, a reduced number of internal coordinates [14, 15] or even adopting more unbiased algorithms recently proposed [16]. In this respect, our group has developed [17] and applied [18, 19] a methodology (hereafter briefly termed the Ellipsoid‐Method‐for‐Clusters‐in‐Solvent, EMCS) which, starting from an MD simulation of the chromophore of interest (hereafter S) embedded in N solvent molecules (SOLV) allows us to unambiguously identify, and then extract, a subtrajectory of a number M<N of SOLV contained within an ellipsoid best fitting the solute S. Making use of the specific features described in detail in the original paper [17] the conformational analysis of such $S{\left(SOLV\right)}_{M}$ cluster *in principle* coincides with the conformational analysis of whatever molecular system along an MD simulation. It follows that the identification of the relevant (transient) $S{\left(SOLV\right)}_{M}$ conformations, as well as their relative stability (statistical weight), can be carried out through whatever well‐assessed cluster‐analysis protocols normally used for analyzing molecular conformational transitions along MD trajectories [20, 21]. For this purpose, in all the EMCS applications, we have so far systematically adopted Essential Dynamics (ED) [22, 23]. The advantage of ED analysis [23] lies in its ability to identify a reduced number, typically not exceeding two or three, of generalized internal degrees of freedom (the Essential Eigenvectors, EEs), allowing a relatively straightforward conformational analysis even in complex molecular structures. However, when the number of EEs turns out to be larger than three, the conformational analysis could become very problematic from a practical point of view. This might indeed occur when ED is applied in conjunction with EMCS. In these cases, in fact, the number of EEs can rapidly become prohibitively large as the strength of the solute–solvent interaction decreases and/or the number of solvent molecules increases [18, 19].

**FIGURE 1:**
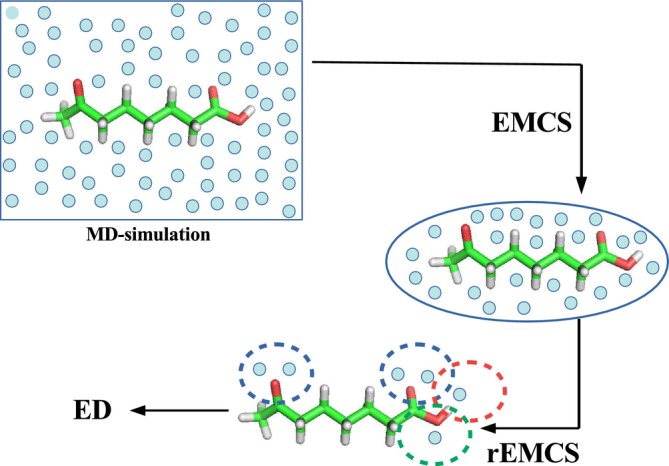
Schematic representation of the sequential steps for rEMCS. See also the Results section. (i) The EMCS, performed on the MD‐simulation, produces a subtrajectory of the solvent molecules (schematically shown as light‐blue spheres) contained in the ellipsoid best approximating the solute molecule. (ii) A number of the solute atoms, conceivably capable of undergoing tighter interactions (if any) with the solvent molecules, are selected and highlighted with dotted circles. (iii) The solvent molecules falling in these selected regions are taken into account; all the other solvent molecules are removed. (iv) The reduced trajectory is analyzed through ED.

To overcome this possible drawback, we report in this study a simple method to reduce the solvent molecules in the $S{\left(SOLV\right)}_{M}$ extracted through EMCS. Our idea is inspired by the intuitive consideration that, if present, relatively stable solute–solvent microclusters can only be identified where relatively stable physical solute–solvent interactions can be formed, that is, typically at the solute–solvent interface, also termed as *first solvation shell*. Consequently, the proposed method, hereafter simply termed as reduced‐EMCS (rEMCS) and schematically depicted in Figure 1, simply consists in eliminating at each frame of the $S{\left(SOLV\right)}_{M}$ cluster trajectory obtained from EMCS, all the solvent molecules not directly linked to specific parts of the S capable of forming more stable interactions (e.g., polar groups, Lewis acids or bases). This approach, while maintaining the features of EMCS, should allow a simplification of the subsequent ED analysis. To test its validity, we decided in the present study to apply this method to the modeling of Vibrational Circular Dichroism (VCD) spectra on a number of well‐studied systems.

VCD [24, 25, 26, 27, 28, 29] has become, in the last few decades, one of the most powerful tools for determining the absolute configuration of several classes of molecules [30, 31, 32, 33, 34, 35]. A strong impetus to the development of this experimental technique has also been given, in the last years, by the availability of efficient theoretical‐computational models [36, 37, 38] nowadays brought to extremely advanced levels of accuracy [39, 40, 41, 42, 43, 44, 45, 46, 47, 48, 49, 50, 51, 52, 53]. VCD spectral signals in condensed phase are known to depend on the explicit interaction with the solvent [53, 54, 55, 56, 57, 58] as witnessed by several studies, either using the “cluster‐in‐solvent” approach [58, 59, 60, 61, 62, 63, 64, 65, 66, 67] or using purely classical methods, based on MD simulations, exploiting the time‐correlation formalism [68, 69, 70, 71, 72, 73]. Because of this peculiarity, the modeling of VCD spectra appeared as particularly suitable for the main purpose of the present study: To propose a method that allows capturing the morphological characteristics of the solvation shells in a rigorous but, at the same time, simple, effective, and computationally inexpensive way. In this study we selected three well investigated systems: (i) aqueous alanine (ALA) [12], (ii) (1S,2S)‐trans‐1‐amino‐2‐indanol (trans‐AI) in dimethyl sulfoxide [62], and (iii) aqueous capped alanine dipeptide (ADP), Ac‐Ala‐NHMe [70, 74] where the crucial role of the first solvation shell, for the correct VCD modeling, has been widely discussed in the referenced studies. We wish to underline that the success of EMCS and rEMCS strongly depends on the quality of the MD simulations (both force field and length of the trajectory) and on the level of the quantum‐chemical calculation for the spectral observable. All these aspects have not been addressed in this study, whose primary aim was to describe the proposed approach using model systems already addressed by other groups. This study is organized as follows. In the first section (Computational details), after a brief outline of the general computational details, we describe in detail both the basic features and the spirit of the method. Subsequently, the results concerning the VCD spectra of the previously cited selected systems are presented and discussed.

## 2 Computational Details

### 2.1 Details of the Molecular Dynamics Simulations and the Essential Dynamics Analysis

All the MD simulations were carried out using the Gromacs program, version 5.0.4 [75], following the standard common protocol described below. An initial energy minimization of the whole box was first carried out. The system was then gradually heated from 50 K to the temperature of interest (300 K, if not otherwise stated) using short (200 ps) MD simulations. The simulation was propagated for the productive run (see Results section) in the NVT (constant number of molecules, volume, and temperature) ensemble using the Velocity rescaling algorithm for keeping the temperature constant [76]. The isobaric conditions in the NVT simulations were mimicked using a methodology recently proposed by our laboratory, based on adjusting the box size of the solute–solvent system to reproduce the average pressure previously obtained by simulating the same number of solvent molecules at the same temperature and at the liquid density corresponding to 1.0 bar. The LINCS algorithm was used to constrain all bond lengths [77]. Long‐range electrostatics were computed by the Particle‐Mesh Ewald method [78] using 34 wave vectors in each dimension and a 4th‐order cubic interpolation. In all the simulations the CHARMM force field [79] and the SPC model [80] were used for the solutes and the solvent, respectively. Additional information, different for each of the investigated systems, is reported in the Results section. The ED analysis was performed as follows. At each frame of the simulation, the coordinates—or a subset of coordinates (see Results section)—of the species of interest were roto‐translationally fitted to a reference structure to include only the “internal” degrees of freedom in the analysis. The corresponding covariance matrix was then constructed and diagonalized, providing a set of orthonormal eigenvectors and their corresponding eigenvalues—the former representing the eigendirections along which the fluctuation of the species of interest can be described, and the latter representing the extent of the fluctuations. The eigenvectors showing the largest eigenvalues, that is, the EEs, provide the subset of internal coordinates usable to follow the conformational transitions of the species of interest [22, 23]. In all the investigated systems, the convergence of the results was ascertained by repeating the ED‐based conformation analysis on three sub‐trajectories (portions) of the MD trajectories obtained from the rEMCS step. In all the cases, the results turned out to be virtually coincident. Additional details can be found in the Supporting Information (SI), Section S.5. Finally, a quantitative estimation of the agreement with experimental data was carried out by calculating the Root Mean Square Deviation (RMSD) of the experimental and calculated spectra as reported in detail in the SI (Section S.6). Note that this was accomplished by using the normalized intensities of both the experimental and the calculated spectra.

### 2.2 Details of Quantum Chemical Calculations

The VCD spectra of all the extracted solute–solvent clusters were calculated in the framework of the Density Functional Theory (DFT) according to a common protocol described below. First, a constrained minimization was carried out for solute–solvent clusters extracted upon rEMCS. This was accomplished by freezing the following solute–solvent (internal) coordinates: (i) all the solute torsion angles (if any), (ii) the solvent molecules' center of mass in spherical coordinates defined with respect to the solute center of the mass, (iii) the torsion angles associated with the solvent rigid‐body rotations with respect to the solute molecule and, finally, (iv) the solvent molecules' internal torsion angles (if any). The constrained‐minimized structures $S{\left(SOLV\right)}_{M}$ were used for calculating the corresponding VCD spectra according to the standard procedures [36, 38] as implemented in the Gaussian16 software [81]. Each spectral line was then fitted to a Gaussian function with a bandwidth at 1/e peak height [82], the value of which is specified below for each system. The whole spectrum was finally obtained by summing the individual spectra of all the $S{\left(SOLV\right)}_{M}$ conformations, weighted for the corresponding probability ($p_{j,i}$) (see Results section). Additional information, different for each of the investigated systems, is reported in the Results section.

## 3 Results

### 3.1 Outline of the Method

**FIGURE 2:**
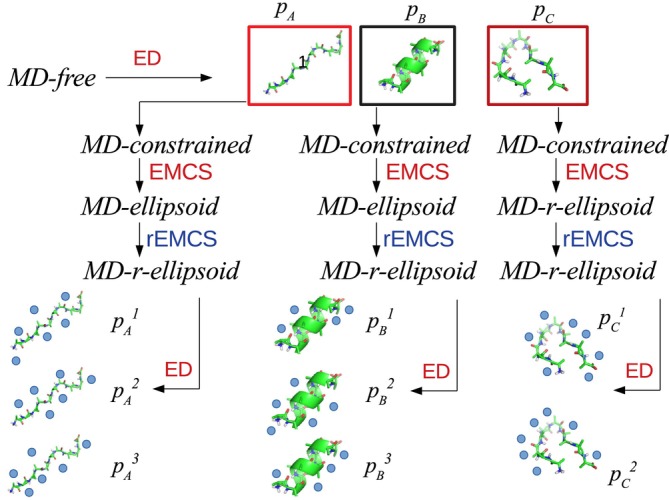
Schematic summary of all the steps for collecting the ensemble of solute–solvent clusters from the rEMCS analysis. With $p_{i}$ we indicate the probability of each solute conformational state as obtained from MD−free. With $p_{j}^{i}$ we indicate the probability of each cluster conformational state as obtained from *MD‐constrained*/EMCS/*MD‐ellipsoid*/rEMCS/*MD‐r‐ellipsoid*/ED sequence. The water molecules are reported as blue spheres for the sake of clarity.

In this preliminary section, we describe the sequence of all the steps, also pictorially represented in Figure 2, that make up the rEMCS computational strategy.

- *Step 1. MD‐free simulation and solute conformational analysis*.
  The first step—common to all the cluster‐in‐solvent‐based approaches—is aimed at determining the conformational space of the solute. This step, unnecessary if the solute is a rigid or semirigid molecule or if the solute conformational repertoire is well assessed (see below in the case of ADP), can be performed through an MD (or even MC) simulation of the whole system, hereafter termed *MD‐free*, followed by standard methods of analysis such as the distribution of internal coordinates or ED (see S.I., Section S1 for details). As a result, a number of solute representative conformations with their statistical weights, $p_{i}$, are identified.
- *Step 2. MD‐constrained simulation and the Ellipsoid‐Method‐for‐Clusters‐in‐Solvent application*.
  Each of the representative conformations extracted in the previous step is then independently simulated (see Figure 2) using the same box, number of solvent molecules, and protocol adopted for *MD‐free*. In these new trajectories, termed as MD−constrained, the coordinates of the j‐th representative conformation are kept frozen (restrained), with only the solvent molecules allowed to move, to reduce as much as possible the size of the conformational space, which must now necessarily include the solvation shells closer to the solute. For this purpose, from the application of EMCS to each of these MD−constrained, we obtain as many subtrajectories, hereafter termed as *MD‐ellipsoid*, containing only the $S{\left(SOLV\right)}_{M}$ cluster with the frozen solute (S) and the M solvent (SOLV) molecules (see Figure 2). If the solute is not small enough [17], the number M of solvent molecules can become too large, and the corresponding ED analysis (see S.I. Section S1 for additional details) would suggest that an excessively large number of EEs would be necessary to analyze the solvent conformational space. The analysis thus becomes intractable, and in these cases, a simplification through rEMCS could be important.
- *Step 3. Ellipsoid reduction (rEMCS) and subsequent ED analysis*.
  Following the scheme depicted in Figure 2, at each frame of the *MD‐ellipsoid*, using the same criterion followed for EMCS [17], we select a subset of solvent molecules conceivably defining the minimal solvation shell. The general strategy for this purpose, as already concisely represented in Figure 1, consists of selecting n solvent molecules interacting with each of the preselected l atoms of the solute, supposed—if any—to undergo tighter interactions with the solvent. Obviously, both n and l are arbitrary parameters that must be selected on the basis of the chemical features of the structure under study. The outcome of the rEMCS is, then, a new trajectory hereafter termed as *MD‐r‐ellipsoid*, containing the k = n x l solvent molecules and the solute. Note that the result is virtually coincident with what could be obtained by defining as many statistically correlated ellipsoids (and as many as the EMCS) as the selected l aggregation centers. The ED analysis, carried out on this *MD‐r‐ellipsoid*, would allow the extraction of the $S{\left(SOLV\right)}_{k}$ representative configurations, whose statistical weight $p_{i}^{j}$, should now take into account the statistical weight of the solute i−th basin and the $S{\left(SOLV\right)}_{k}$ j−th basin which are then used for calculating the spectral observable through Quantum Chemical calculations, as explained in the Computational Details Section and explicitly reported in the three following subsections.

### 3.2 VCD Spectrum of Aqueous L‐Alanine

**FIGURE 3:**
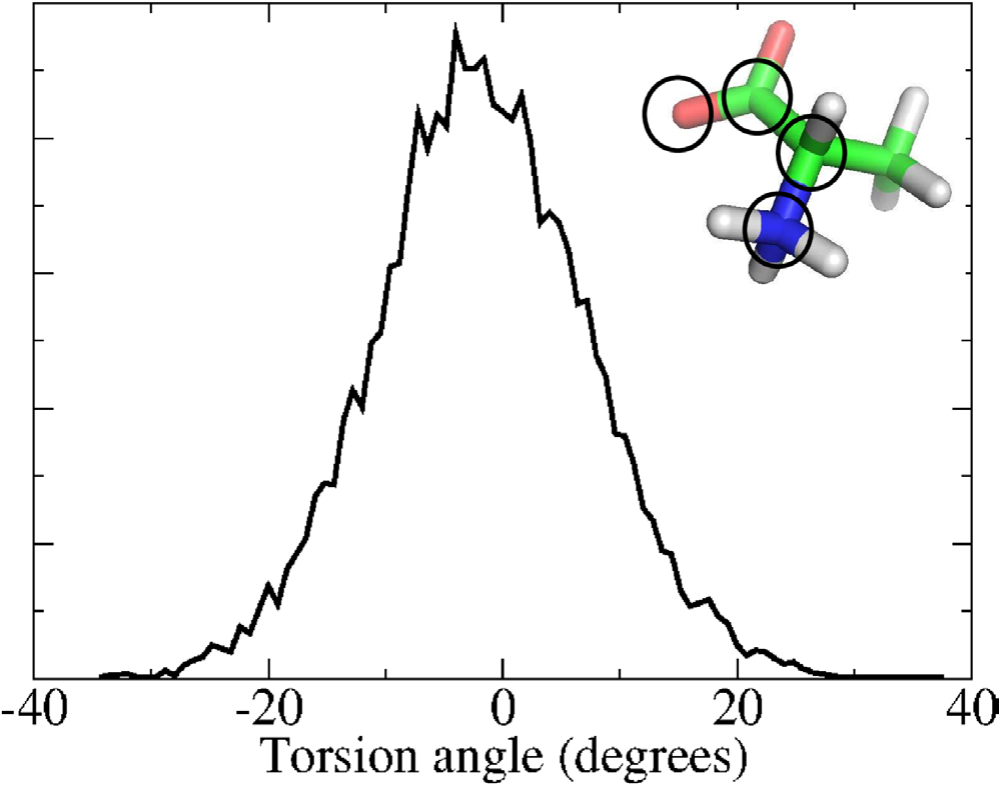
Distribution of the L‐ALA torsion angle (shown in the inset) sampled along the 100 ns of the MD−free simulation.

The theoretical modeling of the VCD spectrum of aqueous L‐Alanine (L‐ALA) has been at the center of several studies, and it is now widely accepted that the explicit water distribution around the chiral solute significantly affects the VCD spectral features [83, 84]. Recently, Canuto and coworkers showed that, for a satisfactory agreement with the experimental spectrum, it is not only necessary to include, in the Quantum‐Chemical calculations, the explicit water molecules of the first solvation shell, but it is also very important to take into account a huge number of solute–solvent‐cluster structures, as obtained from MD simulations, mimicking what the authors call “thermal disorder” [12]. The sequential application of EMCS and rEMCS, followed by ED, as described in this section, reveals that the same level of accuracy can be reached by limiting the Quantum‐Chemical calculations to a reduced number of *essential* microclusters, properly taking into account the water *first solvation shell*. The simulated system consisted of one L‐ALA molecule inserted in a cubic box filled with 234 water molecules to sample the L‐ALA conformational space. 100 ns of *MD‐free* were produced and analyzed following the torsion angle (the only internal L‐ALA coordinate excluding the conformationally irrelevant methyl and ammonium free rotations). The corresponding distribution, reported in Figure 3 clearly indicates that, as expected [12], in the simulated conditions, the complete rotation of the L‐ALA torsion angle, however irrelevant for our purposes due to its 2‐fold symmetry, occurs on time scales much larger than 100 ns.

**FIGURE 4:**
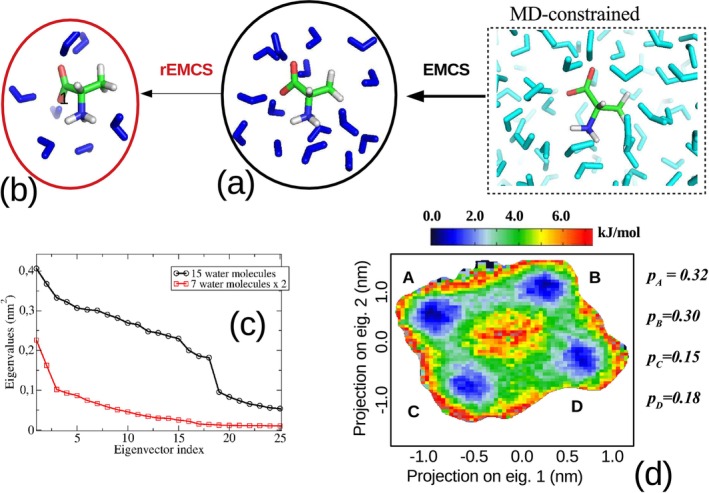
(a) Snapshot of the $L-ALA{\left(H_{2}O\right)}_{15}$ cluster as obtained from the EMCS application to the MD−constrained. (b) Snapshot of the $L-ALA{\left(H_{2}O\right)}_{7}$ cluster as obtained from the application of the rEMCS to the MD−ellipsoid. (c) spectrum of the eigenvalues of the all‐atom covariance matrix of $L-ALA{\left(H_{2}O\right)}_{15}$ (black curve) and $L-ALA{\left(H_{2}O\right)}_{7}$ (red curve) clusters. (d) 2D‐free‐energy landscape of the $L-ALA{\left(H_{2}O\right)}_{7}$ cluster. Note that in all these simulations, L‐ALA was kept frozen.

**FIGURE 5:**
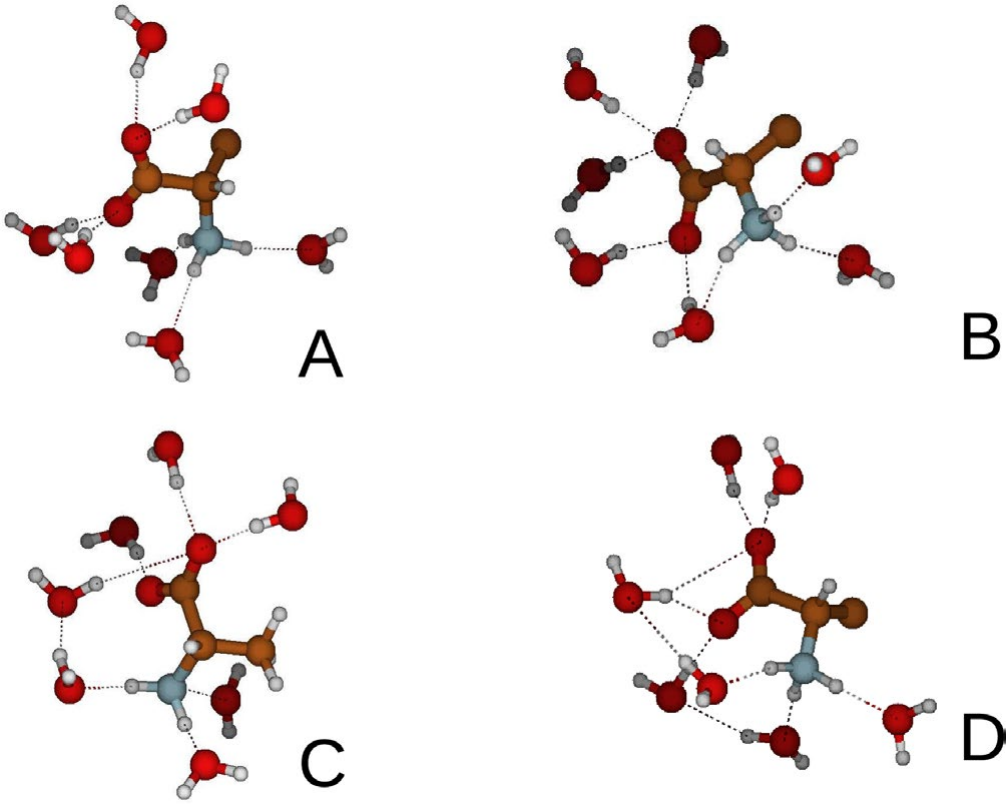
Pictorial view of the representative conformations of the $L-ALA{\left(H_{2}O\right)}_{7}$ cluster of the four basins reported in Figure 4d.

For this reason, and according to the protocol described previously (Figure 2), we performed an MD−constrained simulation, using the same conditions as the MD−free, keeping L‐ALA frozen in the conformation corresponding to the maximum of the distribution reported in Figure 3. Application of EMCS on the MD−constrained produced the MD−ellipsoid subtrajectory of $L-ALA{\left(H_{2}O\right)}_{15}$ cluster (Figure 4a). The corresponding spectrum of the eigenvalues of the covariance atoms (black curve in Figure 4c) turned out to be not sufficiently steep to allow a relatively straightforward cluster analysis. Upon applying rEMCS by selecting four water molecules close to the carboxylate moiety and three close to the ammonium group, Figure 4b, produced the *MD‐r‐ellipsoid* subtrajectory of $L-ALA{\left(H_{2}O\right)}_{15}$ cluster. In this case, the spectrum of the eigenvalues of the covariance matrix (Figure 4c) allowed the use of only two EEs. The resulting 2D‐free energy landscape (see Section S.1 and Equation S.1 in the S.I.) showed, indeed, four very sharp conformational basins (blue spots in the Inset (d) of Figure 4) corresponding to as many distinct representative conformations reported in Figure 5 and the coordinates of which are collected in the S.I. Section S2.

**FIGURE 6:**
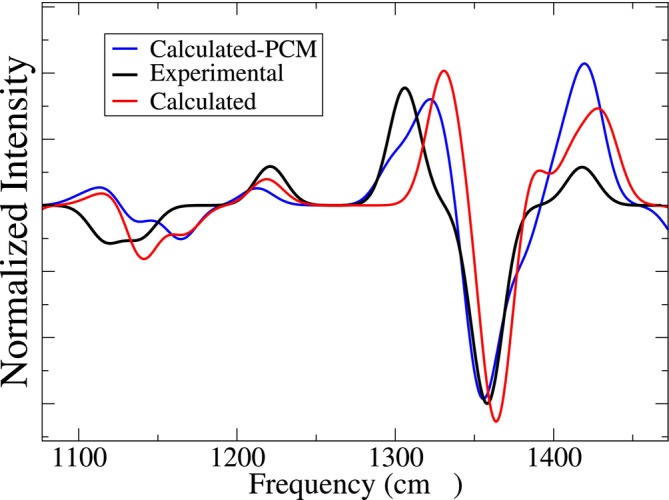
Experimental (black) and calculated (red, and blue with PCM) VCD spectrum of L‐ALA using the individual spectra of the representative conformations reported in Figure 5 and the corresponding probabilities reported in Figure 4d. A bandwidth of 7.0 $cm^{-1}$ was used.

The spectra of each of these representative conformations, calculated as described in the Computational Details section using the B3LYP functional and the 6‐311++G** basis set, were then weighted using the $p_{i}$ reported in Figure 4d and, finally, summed. Additional details of the spectra are reported in the S.I. The final spectrum, depicted in Figure 6 in red, shows a deviation of 10.8 (RMSD, see SI, Section S.6) with respect to the experimental spectrum [85, 86]. The main discrepancies are essentially concentrated in the region between $1,300cm^{-1}$ and $1,400cm^{-1}$, where a significant blue shift of the two main peaks is observed, and also in the overestimation of the relative intensity of the peak at $1,418cm^{-1}$. For this reason, we decided to test the effect of the inclusion of mean‐field by means of the Polarizable Continuum Model (PCM) [87]. The result, reported in the same figure in blue, shows a slight but significant shift to the blue, improving the result in the 1,300–$1,400cm^{-1}$ region. Nonetheless, other less positive effects, see for example the further increase of the signal beyond $1,400cm^{-1}$, should be remarked, and the whole RMSD (14.9) turned out to be slightly higher than that obtained without PCM. In conclusion, our result is in good agreement with the findings of Canuto and coworkers, and confirms that, in the present case, the sequential application of EMCS‐rEMCS‐ED allows for properly capturing the essential features of the thermal disorder with a significantly reduced number (four) of DFT calculations.

### 3.3 VCD Spectrum of (1S,2S)‐Trans‐1‐Amino‐2‐Indanol in Dimethyl Sulfoxide

**FIGURE 7:**
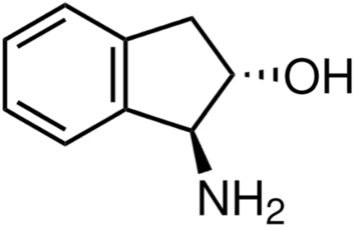
Schematic view of (1S,2S)‐trans‐1‐amino‐2‐indanol.

The second system examined in this study is (1S,2S)‐trans‐1‐amino‐2‐indanol (trans‐AI, see Figure 7) solvated in dimethyl sulfoxide (DMSO), whose VCD spectrum was recently reported by Zehnacker and coworkers [62]. They emphasized the crucial role of explicitly including DMSO molecules for accurate and reliable modeling. Trans‐AI is characterized by three semiclassical large‐amplitude internal degrees of freedom (i.e., alicyclic ring puckering, hydroxyl and amino group free‐rotations) able, in principle, to produce a relatively high number of quasi‐degenerate conformations [62]. For this reason, unlike the system presented in the previous paragraph—characterized by a single well‐defined conformational internal coordinate—we employed ED for the preliminary conformational analysis of trans‐AI. Following the protocol outlined in Figure 2, we first explored the conformational space of trans‐AI using the MD−free simulation, which was propagated for 60.0 ns. This simulation was performed by placing a single trans‐AI molecule in a cubic box filled with 180 DMSO molecules. The Gromos54a7 force field from the Automated Topology Builder [88] was used for both trans‐AI and DMSO. Additional details can be found in the S.I. (Section S.3).

**FIGURE 8:**
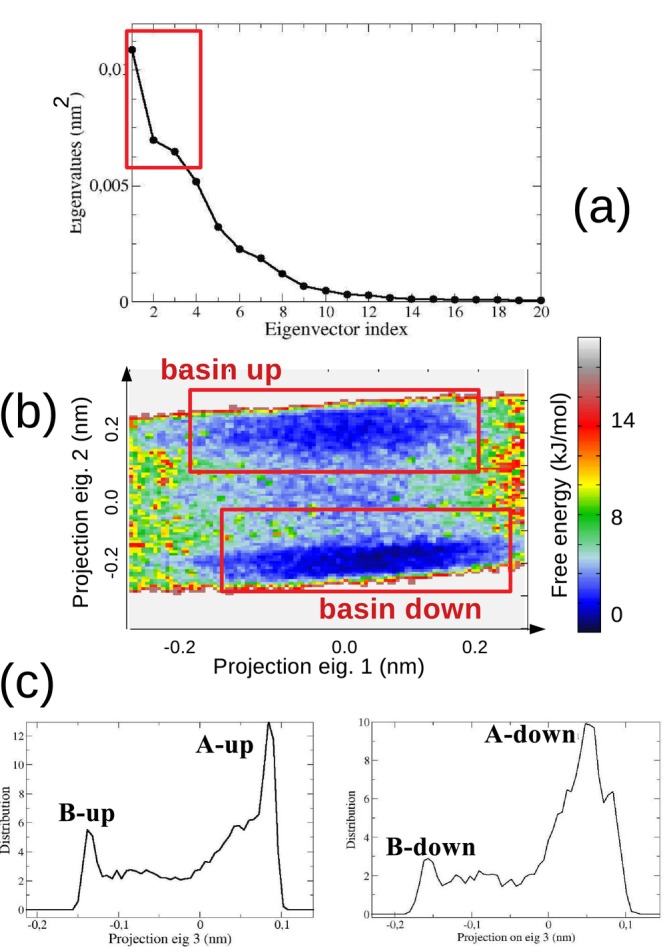
(a) Spectrum of the eigenvalues from the diagonalization of the all‐atom covariance matrix of trans‐AI. The first eigenvector is characterized by concerted hydroxyl rotation and ring puckering; the second and third eigenvectors are characterized by in‐phase and out‐of‐phase rotations of the amino and hydroxyl groups. (b) Free energy (Equation S.1 in the S.I.) landscape in the space of the first two eigenvectors of the all‐atom covariance matrix. (c) Distribution of the projections on the third eigenvector of the all‐atom covariance matrix of the trans‐AI structures along the MD−free.

**FIGURE 9:**
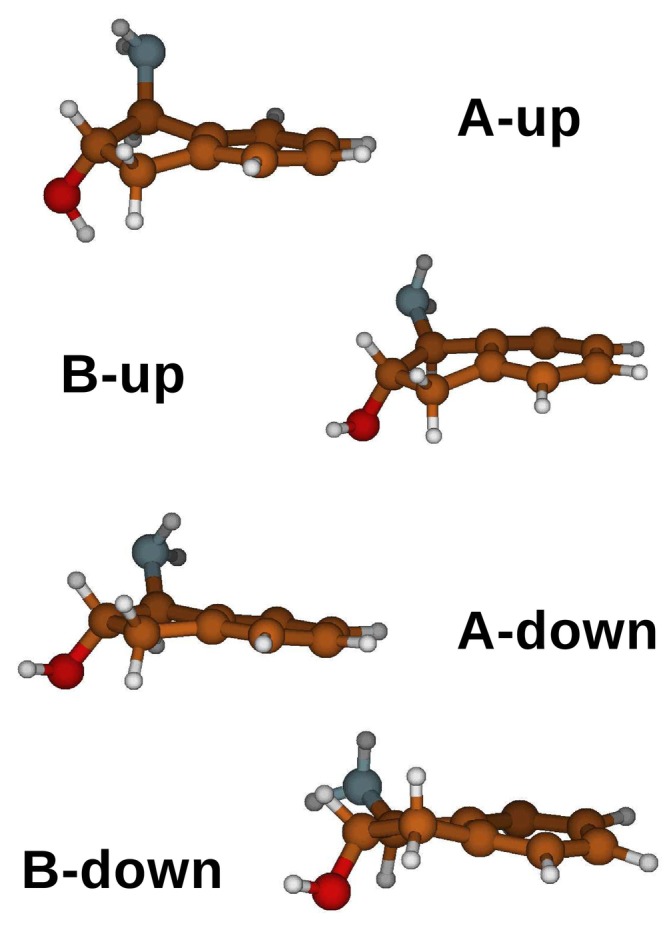
Schematic view of the four structures of trans‐AI extracted from the ED analysis outlined in the Figure 8.

The spectrum of the eigenvalues (inset a of Figure 8) of the all‐atom trans‐AI covariance matrix, as obtained from ED analysis performed along the MD‐free (see Section S.1 of the S.I.), clearly shows at least three EEs. Hence, to properly characterize the trans‐AI conformational repertoire, we should use the first three eigenvectors (highlighted in red in the same figure), which account for more than 60 percent of the whole trans‐AI internal fluctuations. For this purpose, at each frame of the MD−free we first projected the trans‐AI coordinates onto the first two EEs obtaining the free‐energy landscape (see Equation S.1 in the S.I.) depicted in the inset (b) of the Figure 8; the surface is characterized by two well separated conformational basins, indicated as basinup and basindown. Subsequently, all the structures falling in each of the two basins were then separately projected onto the third EE producing the bimodal distributions reported for the basinup and the basindown in the left and right side of the inset (c) of Figure 8, respectively. Four distinct trans‐AI conformational states—corresponding to the maxima of the distributions, were then identified, namely A−up (probability 0.15), B−up (probability 0.25), A−down (probability 0.40), and B−down (0.20), and schematically reported in Figure 9.

**FIGURE 10:**
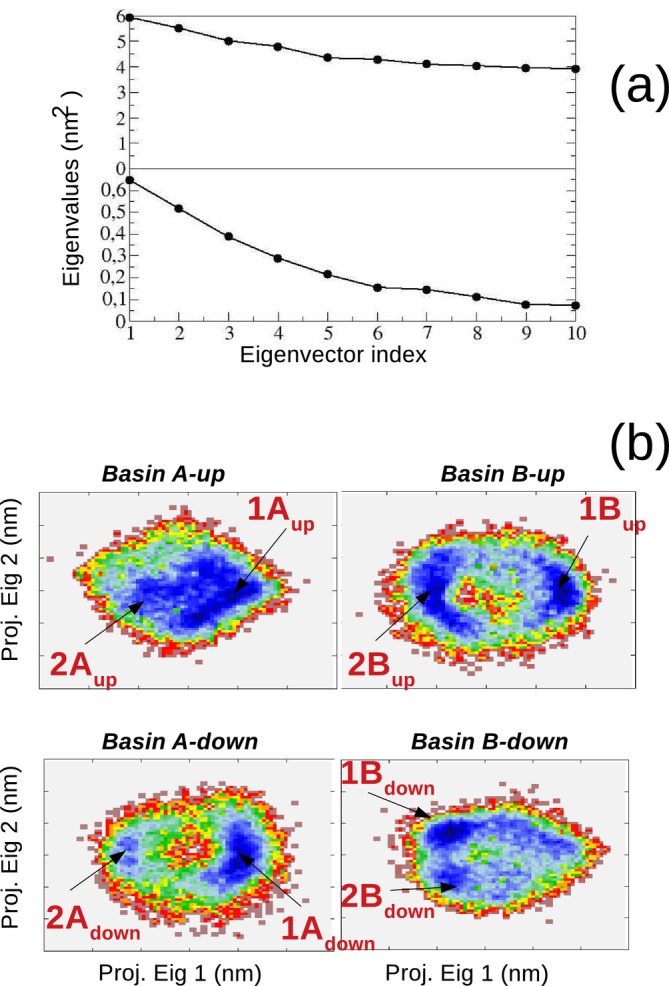
(a) Spectrum of the eigenvalues for $trans-AI{\left(dmso\right)}_{15}$ (top panel) and $trans-AI{\left(dmso\right)}_{3}$ (bottom panel) ellipsoids. (b) Free energy landscapes (Equation S.1 in the S.I.) as obtained from the ED analysis on the four MD−r−ellipsoid simulations.

These structures (see Figure 2) were used to run four independent MD−constrained simulations of 60.0 ns each, following the same protocol and simulation box as the MD−free simulation but keeping trans‐AI frozen in its corresponding conformation, as reported in Figure 9. EMCS analysis of these simulations revealed that 15 DMSO molecules were necessary to properly define the ellipsoid that best approximates trans‐AI. The ED analysis performed on the MD−ellipsoid simulations, as derived from EMCS, exhibited a very flat spectrum with extremely high eigenvalues, as shown in the upper part of panel (a) in Figure 10.

**FIGURE 11:**
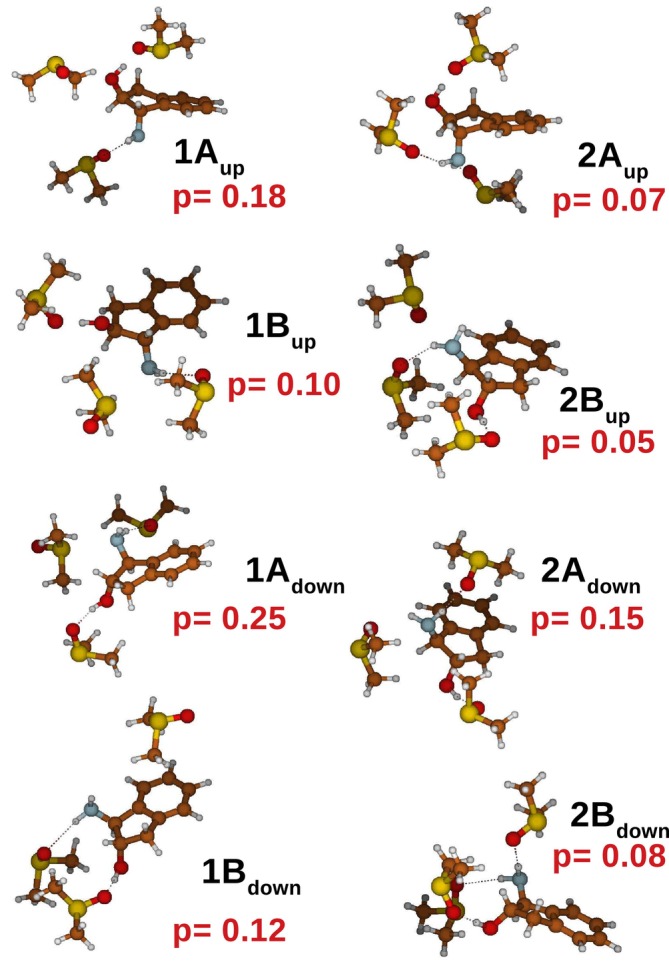
Schematic view of the 8 structures of $trans-AI{\left(dmso\right)}_{3}$ extracted from the ED analysis (see Figure 10) and utilized for the calculation of the VCD spectrum. The corresponding probabilities are reported in red.

This characteristic, essentially due to the very high internal mobility of the DMSO molecules within the solvation shell formed by 15 molecules, made any type of cluster analysis impracticable, necessitating the adoption of rEMCS. Therefore, we decided to focus the analysis on only three DMSO molecules potentially capable of forming H‐bonds with specific moieties of trans‐AI, that is, the hydroxyl and the amino group. The ED analysis, carried out on the resulting MD−r−ellipsoid simulations, shows a significant reduction in the conformational space (see panel (a) in Figure 9), which can be analyzed by considering only two EEs. By projecting on these EEs the $trans-AI{\left(dmso\right)}_{3}$ clusters sampled along the four MD−r−ellipsoid trajectories, we obtained the free‐energy conformational landscapes, reported in panel (b) of the same figure, from which it was possible to identify eight distinct $trans-AI-dmso_{3}$ clusters, indicated in Figure 11 along with their probabilities. These structures were finally utilized to calculate the VCD individual spectra, with details provided in the S.I.

**FIGURE 12:**
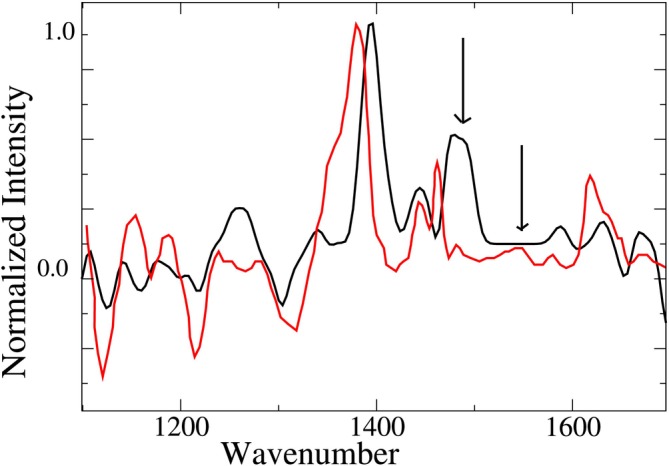
Calculated (black) VCD spectrum using the structures of trans‐AI, and corresponding probabilities reported in Figure 11, compared to the experimental one (red) [62].

The calculated VCD spectrum, reported in Figure 12, satisfactorily parallels the experimental one (RMSD equal to 8.0) with marked disagreements in correspondence of specific signals—either underestimated or overestimated—indicated by arrows. Neither the use of the PCM, as in the previous case, nor the use of a different basis set (data not shown) improved the result, for which a revision of the force field is perhaps necessary.

### 3.4 Aqueous Di‐Alanine Amide‐III Band

The second example reported in this study is the VCD spectrum of aqueous capped di‐Alanine (Ac‐Ala‐NHMe, ADP), with a particular focus on the Amide‐III band, which has been specifically identified as a probe for detecting the conformational states adopted by ADP in water [74]. Also, for this system, the explicit inclusion of the water first solvation shell has been demonstrated to be essential for a correct modeling of spectral observables, either IR or VCD [9, 74]. Moreover, for a relatively flexible species like ADP, it is crucial to carefully consider the corresponding conformational repertoire of ADP and its related contribution to the actual spectral observables. In this respect, experimental [89] and theoretical studies [90] have well assessed that the population of aqueous ADP is dominated by polyproline II ($P_{II}$, about 60% of the whole population) followed by beta (β about 30% of the whole population) and right handed alpha ($\alpha_{R}$) conformations. For this system, we then decided to skip the production of the MD−free preliminary trajectory, instead using the well‐established ADP conformational population and starting our study with the MD−constrained independent simulations of the three constrained conformations. For all these simulations, we used one ADP molecule inserted into a cubic box filled with 5764 water molecules. The three simulations were extended up to 40.0 ns and independently analyzed to extract the solvation shell clusters. For this purpose, according to the general scheme previously outlined, we carried out the sequential EMCS and rEMCS procedures, which in this case allowed us to reduce the size of the solvation shell from 30 to 8 water molecules. We wish to remark that EMCS analysis carried out on the two halves of the MD−constrained simulations produced the same result, hence indicating the actual convergence of the solvent conformational sampling.

**FIGURE 13:**
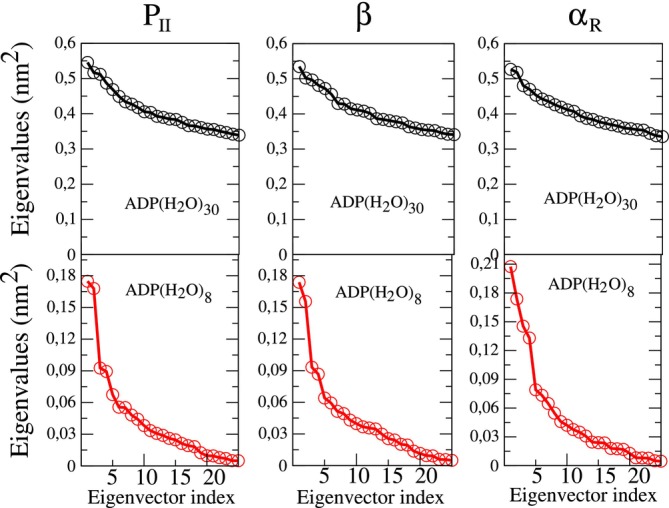
Upper side: Spectra of the eigenvalues of the all‐atom covariance matrix from ED analysis carried out onto the $ADP{\left(H_{2}O\right)}_{30}$ cluster trajectory as emerged from EMCS on the $P_{II}$, β and $\alpha_{R}$ MD−constrained simulations. Lower side: Spectra of the eigenvalues of the all‐atom covariance matrix from ED analysis carried out onto the $ADP{\left(H_{2}O\right)}_{8}$ cluster trajectory as emerged from the rEMCS on the $P_{II}$, β and $\alpha_{R}$ *MD‐ellipsoid* simulations from the previous step.

**FIGURE 14:**
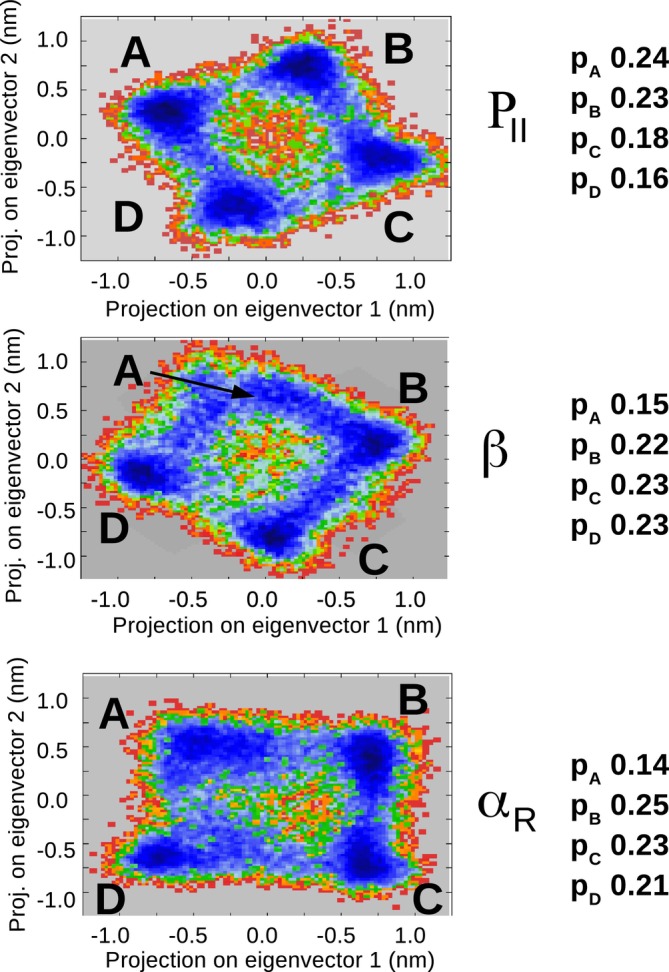
Free energy landscape as a function of the projection of the $ADP{\left(H_{2}O\right)}_{8}$ coordinates onto the first two eigenvectors of the all‐atom covariance matrix along the *MD‐r‐ellipsoid* of $P_{II}$, β and $\alpha_{R}$. The statistical weights are also reported on the right side of the graphs.

**FIGURE 15:**
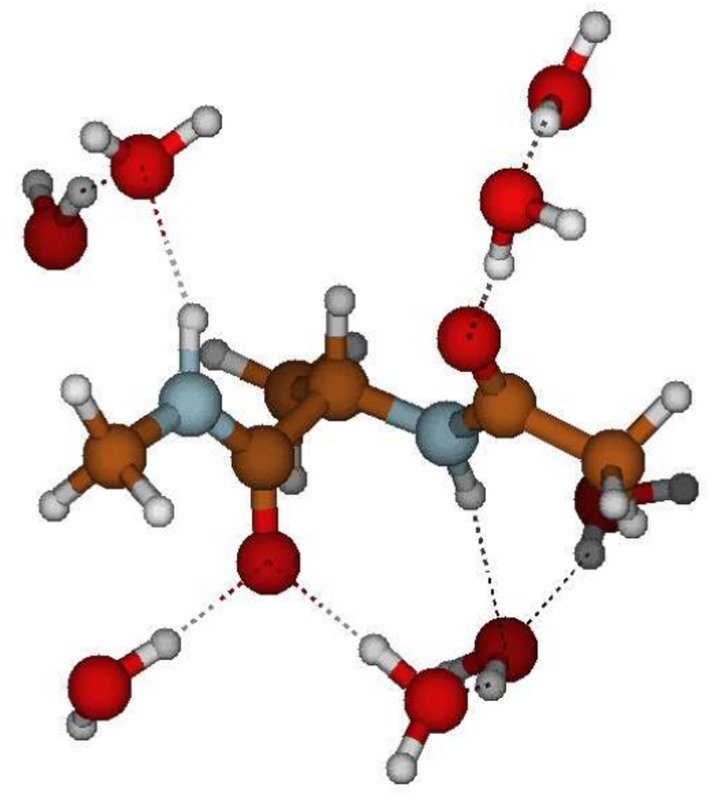
Pictorial representation of the representative conformation for basin A from the ED analysis of the $ADP{\left(H_{2}O\right)}_{8}$ *MD‐r‐ellipsoid* analysis (see Figure 14).

**FIGURE 16:**
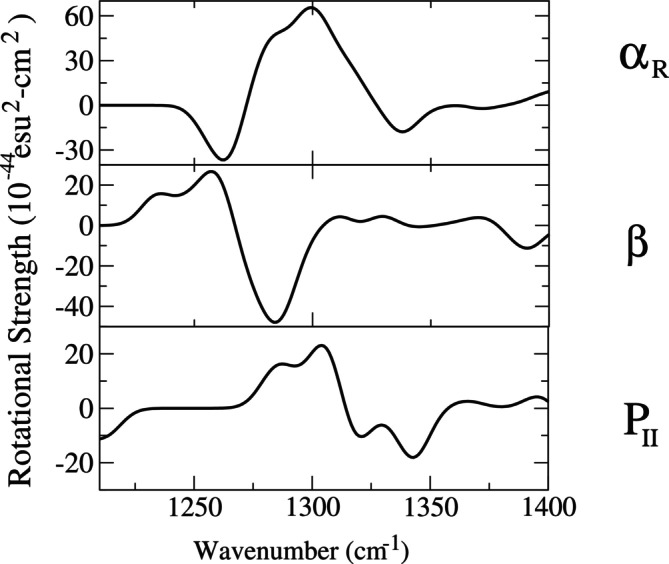
Individual VCD spectra of ADP for $P_{II}$, β, and $\alpha_{R}$ as obtained by summing the spectra of the corresponding representative conformations VCD spectra using the statistical weights reported in Figure 14. A bandwidth of 7.0 $cm^{-1}$ was used.

As shown in Figure 13, the passage from $ADP{\left(H_{2}O\right)}_{30}$ to $ADP{\left(H_{2}O\right)}_{8}$ drastically reduced the dimension of the (essential) conformational space allowing the use of the first two EEs for a proper representation of the $ADP{\left(H_{2}O\right)}_{8}$ conformational analysis. From the resulting free energy landscapes, reported in Figure 14, we could easily identify four (relatively) sharp free energy basins of the $ADP{\left(H_{2}O\right)}_{8}$ cluster for each of the ADP conformations. From these basins, we then extracted the corresponding representative conformations, one of which is reported in Figure 15, that is, corresponding to one of the $P_{II}$. Additional details, that is, the Cartesian coordinates and figures concerning all the other representative conformations, are reported in the S.I. Each of these representative conformations was finally used to model the corresponding VCD signals (see S.I. for details) at the B3LYP/6‐31+G* level. The individual spectra, also reported in the S.I., were eventually merged using their weights ($p_{i}$, Figure 14) to obtain the signals for the three conformations shown in Figure 16. These signals successfully reproduce the characteristic band shape of the amide III region, that is, negative for the β conformation and positive for the other two conformations [74].

**FIGURE 17:**
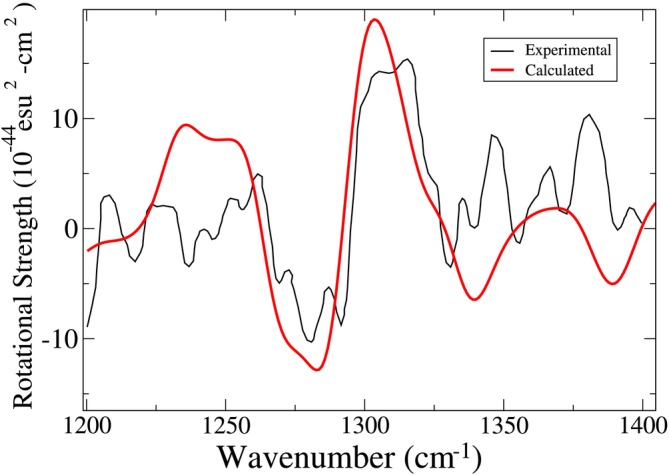
Calculated (red) VCD spectrum of aqueous dialanine, superimposed with a reproduction [85] of the experimental one [86].

The whole spectrum in the amide III region, obtained by summing the signals reported in Figure 16 using the experimental relative population of $P_{II}$, β and $\alpha_{R}$, is reported in Figure 17. Our result, closely similar to the spectrum obtained with a different computational approach [74], correctly reproduces the general experimental characteristics but significantly misses some of the fine structures observed in the red and blue regions. As a matter of fact, the calculated RMSD, order of magnitude higher than the previous cases (see SI, section S.6), testifies to the inability of our approach to reproduce the fine‐structure characterizing (in particular) the red side of the experimental spectrum. In fact, if we restrict the RMSD to the central part of the spectrum (namely 1,260–1,350 $cm^{-1}$), we get a value of 9.6, in line with the corresponding values obtained for the other two systems. In any case, in the present study—specifically aimed at demonstrating the possibility of capturing the essential VCD features with a reduced number of conformations—we did not further investigate this aspect, although it is not irrelevant.

## 4 Conclusions

One decade ago, our group proposed a computational protocol, based on the sequential application of the Ellipsoid‐Method‐for‐Clusters‐in‐Solvent (EMCS) and Essential Dynamics (ED) methods for localizing solute–solvent microclusters as emerged from semiclassical MD simulations. The main, although not the unique, reason that prompted us to propose this method was the need for an unbiased protocol for identifying solute–solvent transient microclusters for the “Cluster‐in‐solvent” approach for computational spectroscopy. However, it was discovered that straightforward applications of the EMCS‐ED were only possible for relatively small solutes in interaction with solvent molecules capable of undertaking strongly localized physical bonds (e.g., H‐bonds). For this reason, we decided to propose a simple evolution of this protocol, termed as Reduced‐EMCS (rEMCS), simply based on the elimination, from the cluster obtained by EMSC, of a number of solvent molecules least interacting with the solute. The sequential application of the EMCS‐rEMCS‐ED on a series of semiclassical MD simulations was then presented in this study. The first reported example, the aqueous 10‐ALA, has shown that even for relatively large solutes with peculiar shapes, rEMCS is able to identify a relatively sharp solute–solvent conformational repertoire. In the other two examples, aqueous L‐alanine and aqueous capped di‐alanine peptide, the validity of the method was also assessed by demonstrating that, with the use of a few number of solute–solvent microclusters, we could reproduce with satisfactory agreement the VCD spectral signals, known to be particularly sensitive to the solute–solvent interaction pattern. rEMCS could then be particularly suitable for highlighting other elusive features of the solvation shells, such as, for example, the chirality transfer currently under study in our laboratory.

## Conflicts of Interest

The authors declare no conflicts of interest.

## Supporting information


**Supplemantary Data S1** (i) Practical steps of the essential dynamics (ED) analysis; (ii) Supplementary results for L‐alanine—B3LYP/6‐311++G**; (iii) Supplementary results for (1S,2S)‐trans‐1‐amino‐2‐indanol; (iv) Supplementary results for Di‐alanine—3LYP/6‐31+G*; (v) Analysis of the convergence of the conformational sampling of the solvent clusters. (vi) Quantitative evaluation of the agreement between calculated and experimental spectra.

## Data Availability

The data that supports the findings of this study are available in the Supporting Information of this article.
