# Supplementary material for: Revisiting the “Cluster‐In‐Solvent” Approach for Computational Spectroscopy: The Vibrational Circular Dichroism as a Test Case
Source: J Comput Chem. 2025 Jun 20;46(17):e70144. doi: 10.1002/jcc.70144 (PMC12179761; doi:10.1002/jcc.70144)
Supplement: Supplementary file 1 — Supplemantary Data S1 (i) Practical steps of the essential dynamics (ED) analysis; (ii) Supplementary results for L‐alanine—B3LYP/6‐311++G**; (iii) Supplementary results for (1S,2S)‐trans‐1‐amino‐2‐indanol; (iv) Supplementary results for Di‐alanine—3LYP/6‐31+G*; (v) Analysis of the convergence of the conformational sampling of the solvent clusters. (vi) Quantitative evaluation of the agreement between calculated and experimental spectra. [file JCC-46-0-s001.pdf]

SUPPLEMENTARY INFORMATION for

Revisiting the ’Cluster-in-solvent’ approach for computational spectroscopy: the Vibrational Circular Dichroism as a test case

Srilatha Arra, Isabella Daidone, Massimiliano Aschi

Index.

S1. Practical steps of the essential dynamics (ED) analysis

S2. Supplementary results for L-alanine – B3LYP/6-311++G**

S3. Supplementary results for (1S,2S)-trans-1-amino-2-indanol

S4. Supplementary results for Di-alanine – B3LYP/6-31+G*

S5. Analysis of the convergence

S6 - Quantitative evaluation of the agreement between calculated and experimental spectra

S1. Practical steps of the essential dynamics (ED) analysis

In this section we describe the steps necessary for determining the free-energy lanscape from ED analysis and, hence, the structure and the statistical weight of the sampled conformations of the system of interest. This procedure, adopted when necessary, for all the systems investigated in this study is described for the case of two Essential Eigenvectors (EEs). In principle the same strategy can be applied for any number of EEs.

Step S1. Once the molecular dynamics (MD) simulation is concluded and the system of interest (hererafter *S*, i.e. a molecule or a cluster in the framework of ECMS or rECMS) is roto-translationally fitted to a reference structure, the *S* covariance matrix is built and diagonalized. The *S* cartesian coordinates are projected, at each frame of the simulation, onto a subset (the EEs) of the obtained eigenvectors showing the largest eigenvalues. This operation produces the figure below (Figure S1) in which each point corresponds to a well defined *S* conformation.

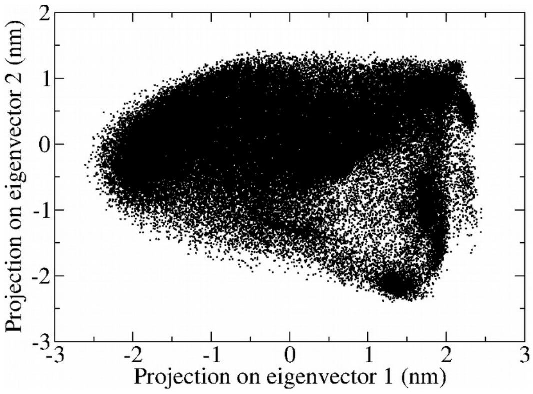

FigureS1. Projection of the cartesian coordinates of the solute onto the first two eigenvectors of the covariance matrix.

Step S2. A M x M grid is generated (Figure S2) for determining a 2D-histogram.

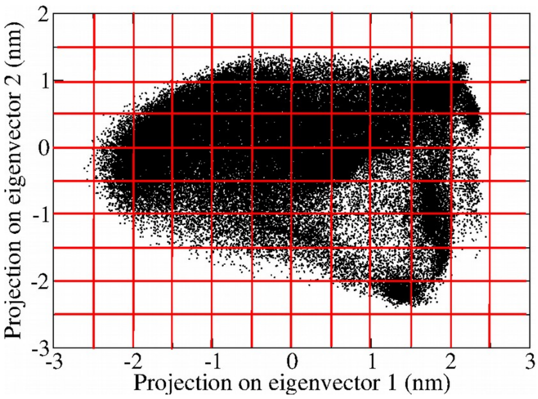

Figure S2. Schematic view of the M x M grid for the 2D-histogram.

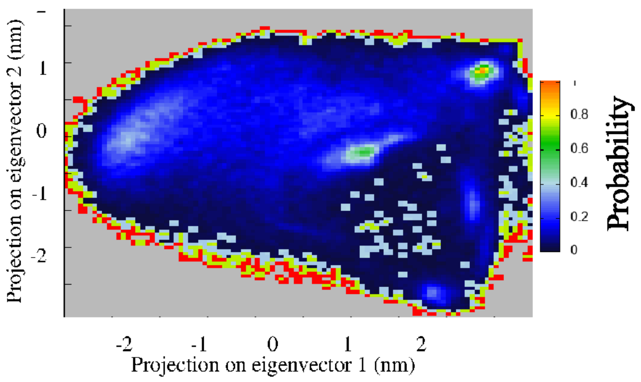

Figure S3. 2D-probability distribution obtained from Figures S1 and S2.

The probability distribution is hence generated considering‘reference’ the square containing the largest number (N_{ref}) of points and defining, for each *i-th* square the corresponding probability P_i as $P_i = N_i/N_{ref}$ obtaining the Figure S3.

Step S3. From each probability we finally can calculate the Helmholtz free-energy lanscape using the standard equation

$$\Delta A_{i\rightarrow j} \simeq -k_B T \ln \frac{P_j}{P_i} \simeq \mu_j^o - \mu_i^o \qquad \text{Equation S.1}$$

In this equation k_B is the Boltzmann constant and P_i and P_j are the probability of two generic squares (or more in general two bins) reported in the Figure S3. Note that the Helmholtz free-energy difference is virtually coincident with the difference of the (standard) chemical potential (μ^o) of the ‘species’ (i.e. conformations) *i* and *j*.

From this equation we obtain the final Figure S4.

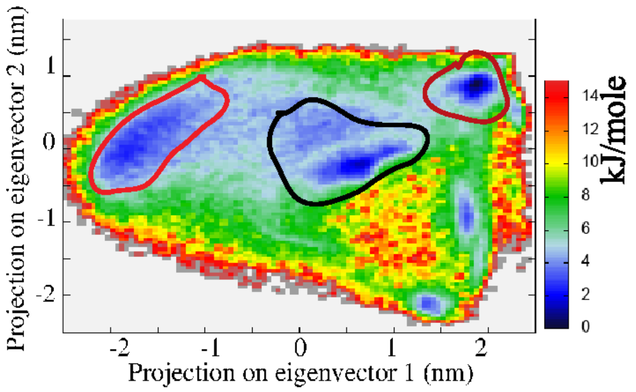

Figure S4. Free-energy landscape from Figure S3 and Equation S1.

The regions corresponding to ‘blue’ spots are indicated as *free-energy basins*. Within each of these basins we can find the collection of all structurally very similar *S* structures, i.e. the S conformations. Hence, from the center of each basin, we extract a single structure which can be considered as *representative* (Representative Conformation) of the whole basin. Obvioulsy, in some

cases, i.e. when the basin is too large, the structural differences (typically the Root Mean Square Deviation) might result too high for allowing the use of ONLY ONE representative conformation. In these cases for each free-energy basin, different representative conformations might be necessary.

S2. Supplementary results for L-alanine – B3LYP/6-311++G**

Cartesian coordinates of the constrained-optimized L-ALA(H_2O)$_7$ clusters from rEMCS analysis carried out onto the *MD-ellipsoid* trajectory in turn obtained from EMCS applied to the initial *MD-constrained* trajectory.

A

6	1.00537	-1.82621	-0.96016
6	0.46114	-0.95908	0.17189
6	0.82817	0.54046	-0.00923
8	2.02991	0.85184	0.11117
7	-1.04176	-0.99606	0.18071
8	-0.14999	1.26166	-0.34531
8	-1.43001	2.86894	-1.90532
8	3.38712	-0.84611	1.70652
8	3.9699	-0.06345	-1.58818
8	-1.59114	-3.70112	0.44777
8	-3.06945	0.50175	1.60242
8	-3.41337	-0.57621	-1.49195
8	-0.11196	2.95745	2.05569
1	-1.36927	-1.96611	0.2881
1	-1.45772	-0.42045	0.92239
1	-1.41447	-0.61829	-0.69872
1	0.81986	-1.31313	1.13962
1	2.09088	-1.73837	-0.99445
1	0.73892	-2.87575	-0.8182
1	0.61487	-1.494	-1.92683
1	-2.30495	-4.1908	0.02439
1	-1.05557	-4.34608	0.92274
1	-2.81602	1.43629	1.58957
1	-3.61079	0.35735	2.38557
1	-3.73694	-0.00989	-2.20037
1	-3.68565	-0.09233	-0.69923
1	-0.87361	2.24281	-1.4063
1	-0.93973	3.60245	-2.29346
1	0.57387	3.61953	2.1751
1	0.06767	2.55275	1.19124
1	2.97793	-0.15866	1.14533
1	4.09073	-1.2182	1.1659
1	4.52393	0.57195	-2.04936
1	3.32207	0.46236	-1.0855

B

6	1.23012	-1.9333	0.35065
6	0.89168	-0.63281	-0.36416
6	-0.40273	0.05067	0.1725
8	-1.47365	-0.59274	0.10321

7	2.03674	0.34458	-0.39147
8	-0.27681	1.25794	0.48836
8	-1.72519	2.90827	-0.70528
8	1.5096	3.07337	-0.25645
8	-3.16639	-0.97942	-1.94926
8	-2.16497	-3.15642	0.79808
8	3.91741	-1.31604	-1.79274
8	-3.85112	0.70385	1.24013
8	3.21364	0.12617	2.31129
1	2.77475	-0.01347	-1.01103
1	1.7528	1.30408	-0.67591
1	2.45878	0.44307	0.54527
1	0.67639	-0.85324	-1.41414
1	0.34838	-2.5758	0.34282
1	2.03828	-2.46658	-0.1552
1	1.52597	-1.75766	1.38751
1	4.34133	-1.34087	-2.65784
1	4.22353	-2.1103	-1.34118
1	0.75705	2.69623	0.23619
1	1.09241	3.73466	-0.82091
1	2.70954	0.54876	3.01723
1	4.13754	0.24245	2.55912
1	-1.44453	2.19118	-0.11144
1	-2.32821	2.43162	-1.28082
1	-3.06602	0.27526	0.86689
1	-3.88198	1.57337	0.83121
1	-2.54661	-0.84186	-1.21981
1	-3.84773	-1.55081	-1.58299
1	-2.29995	-3.19769	1.74907
1	-1.85339	-2.25173	0.62364

C

6	-1.13424	2.10083	0.35461
6	-0.95385	0.75731	-0.36741
6	0.53709	0.33453	-0.25175
8	1.37812	1.03727	-0.83423
7	-1.83798	-0.31023	0.22193
8	0.73575	-0.77532	0.29767
8	2.62906	-1.86772	1.59172
8	2.02201	3.48125	-0.53279
8	-1.70692	0.22201	2.91394
8	-4.13946	0.88016	-0.92363
8	4.12628	0.26344	-0.73137
8	-1.86809	-2.54038	-1.4205
8	0.56682	-2.90041	-0.56573
1	-2.81064	0.01924	0.12629
1	-1.77502	-1.20484	-0.32697
1	-1.61715	-0.45105	1.21898
1	-1.21527	0.84022	-1.42518
1	-0.32344	2.77478	0.06712
1	-2.09096	2.57406	0.10584
1	-1.09815	1.96734	1.43692

1	-4.8713	0.41197	-1.34872
1	-4.45934	1.78458	-0.79797
1	-0.95262	-2.84621	-1.14409
1	-1.76373	-2.22393	-2.32956
1	-2.36191	0.03791	3.60053
1	-0.89522	0.45614	3.38581
1	1.94072	-1.37601	1.1157
1	2.43388	-2.78636	1.3633
1	1.19696	-3.39782	-1.10631
1	0.91446	-1.98298	-0.48167
1	2.87552	3.32209	-0.10762
1	1.78681	2.60271	-0.87131
1	3.17587	0.4631	-0.81122
1	4.13377	-0.37175	0.00323

D

6	-0.67915	-2.10077	-1.17931
6	-0.38634	-1.34262	0.11784
6	0.68423	-0.25476	-0.25859
8	1.81117	-0.67624	-0.57102
7	-1.5539	-0.55937	0.64193
8	0.33349	0.93196	-0.0542
8	4.27007	-0.76114	-1.20952
8	1.83716	2.82111	0.8138
8	-0.69598	2.95444	-1.31148
8	-3.91297	-1.98611	0.40658
8	-2.8382	0.77628	-1.43422
8	2.7878	-2.12602	1.63627
8	-1.20162	1.65817	2.27307
1	-2.32449	-1.13566	0.99092
1	-1.26421	0.1526	1.3361
1	-1.97437	-0.01736	-0.13306
1	0.01418	-1.97243	0.91127
1	0.26506	-2.49246	-1.55572
1	-1.36663	-2.93962	-1.03575
1	-1.10683	-1.428	-1.92484
1	-4.77828	-1.58291	0.27359
1	-4.10507	-2.89272	0.67188
1	-0.32121	2.02481	2.10195
1	-1.42868	1.90918	3.17336
1	-2.34911	1.59939	-1.62419
1	-3.44232	0.63862	-2.16841
1	1.51997	1.97986	0.43099
1	2.79291	2.80218	0.70663
1	0.02161	3.54796	-1.55042
1	-0.24334	2.19622	-0.89692
1	3.3219	-0.55134	-1.18593
1	4.49788	-0.88244	-2.13445
1	2.56316	-1.50079	0.9291
1	3.74526	-2.0903	1.71654

Below we report the VCD signals of the structures previously reported calculated at the same level of theory. Frequencies are reported in cm^{-1}, Rotational strengths are reported in 10^{-44} esu^2cm^2

A

73.4869995	29.2441998
192.649902	15.1426001
207.913300	-0.237399995
228.213104	-19.4706993
236.521606	56.5756989
287.375702	-93.0231018
381.028595	-74.1595993
417.665314	-132.970306
781.481018	77.2657013
846.260010	0.157600001
904.765930	-13.5360003
980.578186	10.7059002
1007.89691	-12.7207003
1109.76355	4.85010004
1139.81982	-44.9799995
1218.35974	20.3894997
1329.03455	82.8245010
1363.76501	-176.637100
1390.53784	105.721901
1427.23975	31.6816998
1497.21375	-33.5582008
1498.85022	1.38090003
1562.18420	-0.536700010
1677.32935	-112.563797
1684.33936	-57.0471992
1710.68213	-146.173004
3035.17114	-1.05050004
3089.58472	-1.35259998
3106.12256	1.96529996
3138.86353	-1.58759999
3313.26245	65.7278976
3387.51587	-75.6511993
3409.10229	44.3935013

B

178.840195	11.8930998
215.999802	81.6572037
226.899002	-14.0254002
245.360596	-13.0017996
403.759888	-23.4535999
411.618103	-50.5705986
419.532104	17.1144009
514.686707	14.8765001
536.026917	282.744293
779.275024	49.0766983
842.730713	0.445100009
913.336670	-9.50119972
1005.55249	-41.6727982

1022.40320	-16.3092995
1123.62000	21.2187996
1166.85315	-44.4211006
1230.67859	2.55159998
1336.74316	65.1468964
1393.24146	-161.962296
1411.02954	82.9738007
1433.06775	83.1676025
1501.75549	-7.52400017
1507.80920	-10.3787003
1570.12024	26.6966991
1660.32495	-278.566711
1700.09387	-18.4027996
1708.74072	-129.871902
1718.97754	77.3627014
3040.37183	2.11969995
3057.14893	20.4314995
3106.30151	-2.91650009
3124.38550	-4.55910015
3153.01538	-69.4296036
3302.14795	-28.4524002
3368.82227	27.7404003

C

72.4150009	-4.95279980
149.179596	2.14700007
204.891296	-36.6128006
218.584503	-2.71370006
263.781006	-60.6809006
274.653503	55.6245003
289.080505	-74.1891022
504.300293	-139.910294
650.328125	35.6509018
765.153625	-86.3945007
854.138184	-7.37109995
924.208496	-29.5750008
1010.75232	2.49379992
1039.99438	-39.6435013
1108.70178	14.6084995
1173.25513	12.0417995
1261.89844	15.8985004
1322.23572	75.6522980
1393.62146	-140.544800
1422.73035	46.9454002
1447.20020	134.061005
1504.49524	-6.09159994
1509.72668	-7.65339994
1580.12927	6.93219995
1627.04773	39.1113014
1666.52588	-430.375885
1721.82324	314.305908
1730.31165	-410.044800

2957.61914	-194.770401
3021.04248	16.2404003
3078.14038	-8.27530003
3093.87305	-31.1117992
3125.93579	9.87839985
3306.86475	188.442398
3349.79932	146.473297

D

217.700500	-18.1401997
231.087097	-31.8302994
416.422791	37.7687988
440.601715	75.4404984
546.093018	-32.9962997
640.433228	107.313202
779.577271	-10.3015003
844.319214	5.00960016
888.446594	-12.1099005
1001.90729	5.01440001
1009.13501	18.9115009
1108.54236	8.62150002
1140.33740	4.65280008
1222.11206	9.71039963
1306.26184	46.4230003
1372.86462	-87.5382996
1398.21155	-9.00080013
1426.74609	52.4249992
1496.45105	-15.9406996
1504.42542	6.01989985
1593.17126	-14.8464003
1628.97632	0.293700010
1658.79492	68.0948029
1710.46204	-476.653503
1738.92505	365.181396
3036.49878	-2.32540011
3098.03979	11.1211004
3118.19409	-4.05569983
3140.16772	2.61910009
3222.37329	-65.6499023
3243.06812	69.6363983
3434.33521	17.9983997

All these signals are reported in the Figure S5 and superimposed with the experimental spectrum amplified for the sake of clarity.

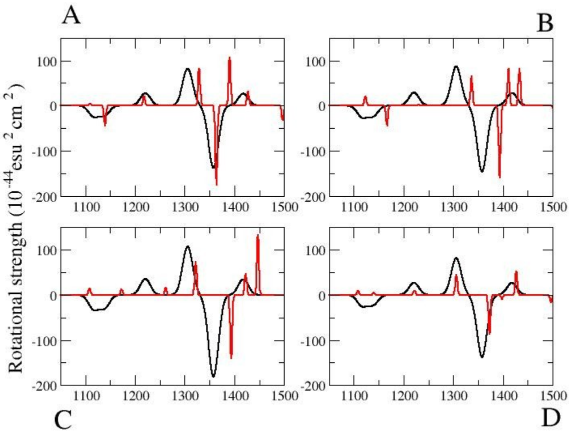

Figure S5. Calculated (red with a Gaussian function with sigma = 2 cm^{-1} centered at the frequency maximum) spectra of L-ALA$(H_2O)_7$ clusters and experimental spectrum (black).

The same figure is reported below with the calculated frequencies shifted in order to match as much as possible the experimental spectrum and using a Gaussian function with sigma = 10 cm^{-1}.

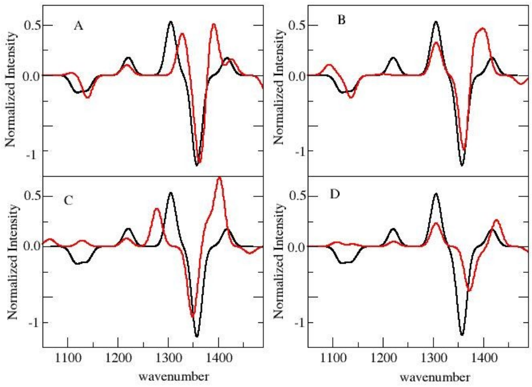

Figure S6. Calculated (red with a Gaussian function with sigma = 10 cm^{-1} centered at the frequency maximum) spectra of L-ALA$(H_2O)_7$ clusters and experimental spectrum (black). The frequencies of the indivisual spectra were red-shifted in order to match as much a spossible the experimental data.

S3. Supplementary results for (1S,2S)-trans-1-amino-2-indanol (Trans-AI)

Gromacs-format topology and atomic charges for trans-AI

```
[ moleculetype ]
; Name   nrexcl
WZZ2     3
[ atoms ]
;  nr  type  resnr  resid  atom  cgnr  charge    mass
   1  HS14    1    WZZ2    H11    1    0.418   1.0080
   2  OAlc    1    WZZ2     O1    2   -0.668  15.9994
   3     C    1    WZZ2     C8    3    0.208  12.0110
   4    HC    1    WZZ2     H8    4    0.021   1.0080
   5  CPos    1    WZZ2     C7    5   -0.163  12.0110
   6    HC    1    WZZ2     H6    6    0.086   1.0080
```

```
    7    HC    1    WZZ2     H7    7    0.087   1.0080
    8  CAro    1    WZZ2     C3    8    0.039  12.0110
    9  CAro    1    WZZ2     C2    9   -0.105  12.0110
   10  CPos    1    WZZ2     C9   10    0.357  12.0110
   11    HC    1    WZZ2     H1   11    0.031   1.0080
   12  NPri    1    WZZ2     N1   12   -0.989  14.0067
   13  HS14    1    WZZ2     H9   13    0.368   1.0080
   14  HS14    1    WZZ2    H10   14    0.368   1.0080
   15  CAro    1    WZZ2     C1   15   -0.050  12.0110
   16    HC    1    WZZ2     H5   16    0.098   1.0080
   17  CAro    1    WZZ2     C6   17   -0.208  12.0110
   18    HC    1    WZZ2     H4   18    0.142   1.0080
   19  CAro    1    WZZ2     C5   19   -0.109  12.0110
   20    HC    1    WZZ2     H3   20    0.127   1.0080
   21  CAro    1    WZZ2     C4   21   -0.191  12.0110
   22    HC    1    WZZ2     H2   22    0.133   1.0080
; total charge of the molecule:  -0.000
[ bonds ]
;  ai   aj  funct   c0         c1
    1    2    2   0.0972   1.9581e+07
    2    3    2   0.1430   8.1800e+06
    3    4    2   0.1100   1.2100e+07
    3    5    2   0.1540   4.0057e+06
    3   10    2   0.1560   3.0819e+06
    5    6    2   0.1100   1.2100e+07
    5    7    2   0.1090   1.2300e+07
    5    8    2   0.1520   5.4300e+06
    8    9    2   0.1400   8.5400e+06
    8   21    2   0.1390   8.6600e+06
    9   10    2   0.1520   5.4300e+06
    9   15    2   0.1390   8.6600e+06
   10   11    2   0.1100   3.0579e+06
   10   12    2   0.1470   8.7100e+06
   12   13    2   0.1020   1.7782e+07
   12   14    2   0.1020   1.7782e+07
   15   16    2   0.1090   1.2300e+07
   15   17    2   0.1400   8.5400e+06
   17   18    2   0.1090   1.2300e+07
   17   19    2   0.1390   8.6600e+06
   19   20    2   0.1090   1.2300e+07
   19   21    2   0.1400   8.5400e+06
   21   22    2   0.1090   1.2300e+07
[ pairs ]
;  ai   aj  funct  ;  all 1-4 pairs but the ones excluded in GROMOS itp
    1    4    1
    1    5    1
    1   10    1
    2    6    1
    2    7    1
    2    8    1
    2    9    1
    2   11    1
    2   12    1
    3   13    1
    3   14    1
    3   15    1
    3   21    1
    4    6    1
    4    7    1
    4    8    1
    4    9    1
    4   11    1
    4   12    1
```

```
    5   11    1
    5   12    1
    5   15    1
    5   19    1
    5   22    1
    6    9    1
    6   10    1
    6   21    1
    7    9    1
    7   10    1
    7   21    1
    8   11    1
    8   12    1
    8   16    1
    8   20    1
    9   13    1
    9   14    1
    9   18    1
    9   22    1
   10   16    1
   10   17    1
   10   21    1
   11   13    1
   11   14    1
   11   15    1
   12   15    1
   15   20    1
   16   18    1
   16   19    1
   17   22    1
   18   20    1
   18   21    1
   20   22    1
[ angles ]
;  ai   aj   ak  funct   angle     fc
    1    2    3    2    109.50   450.00
    2    3    4    2    110.30   524.00
    2    3    5    2    111.00   530.00
    2    3   10    2    115.00   610.00
    4    3    5    2    108.53   443.00
    4    3   10    2    107.60   507.00
    5    3   10    2    109.50   520.00
    3    5    6    2    108.53   443.00
    3    5    7    2    113.00   545.00
    3    5    8    2    106.00  1733.55
    6    5    7    2    107.57   484.00
    6    5    8    2    111.00   530.00
    7    5    8    2    113.00   545.00
    5    8    9    2    111.00   530.00
    5    8   21    2    132.00   760.00
    9    8   21    2    120.00   560.00
    8    9   10    2    111.00   530.00
    8    9   15    2    120.00   560.00
   10    9   15    2    126.00   640.00
    3   10    9    2    106.00  1733.55
    3   10   11    2    106.75   503.00
    3   10   12    2    120.00   560.00
    9   10   11    2    109.50   448.00
    9   10   12    2    115.00   610.00
   11   10   12    2    106.75   503.00
   10   12   13    2    109.50   425.00
   10   12   14    2    109.50   425.00
   13   12   14    2    106.75   503.00
```

```
   9   15   16    2    120.00   505.00
   9   15   17    2    120.00   560.00
  16   15   17    2    120.00   505.00
  15   17   18    2    120.00   505.00
  15   17   19    2    120.00   560.00
  18   17   19    2    120.00   505.00
  17   19   20    2    120.00   505.00
  17   19   21    2    120.00   560.00
  20   19   21    2    120.00   505.00
   8   21   19    2    120.00   560.00
   8   21   22    2    120.00   505.00
  19   21   22    2    120.00   505.00
[ dihedrals ]
; GROMOS improper dihedrals
;  ai   aj   ak   al  funct   angle     fc
  15    9   16   17    2      0.00   167.36
   9    8   10   15    2      0.00   167.36
   8    5    9   21    2      0.00   167.36
  21    8   19   22    2      0.00   167.36
  19   17   20   21    2      0.00   167.36
  17   15   18   19    2      0.00   167.36
[ dihedrals ]
;  ai   aj   ak   al  funct    ph0      cp     mult
   1    2    3    5    1      0.00     1.26    3
   3    5    8    9    1      0.00     1.00    6
   5    3   10    9    1    180.00     1.00    3
   8    9   15   17    1    180.00    41.80    2
   9    8   21   19    1    180.00    41.80    2
   9   10   12   13    1    180.00     1.00    6
   9   15   17   19    1    180.00    41.80    2
  10    3    5    8    1    180.00     1.00    3
  15    9   10    3    1      0.00     1.00    6
  15   17   19   21    1    180.00    41.80    2
  17   19   21    8    1    180.00    41.80    2
  21    8    9   15    1    180.00    41.80    2
[ exclusions ]
;  ai   aj  funct  ;  GROMOS 1-4 exclusions
   8   17
   9   19
  15   21
```

Composition of the three Essential Eigenvectors from diagonalization of all-atom trans-AI

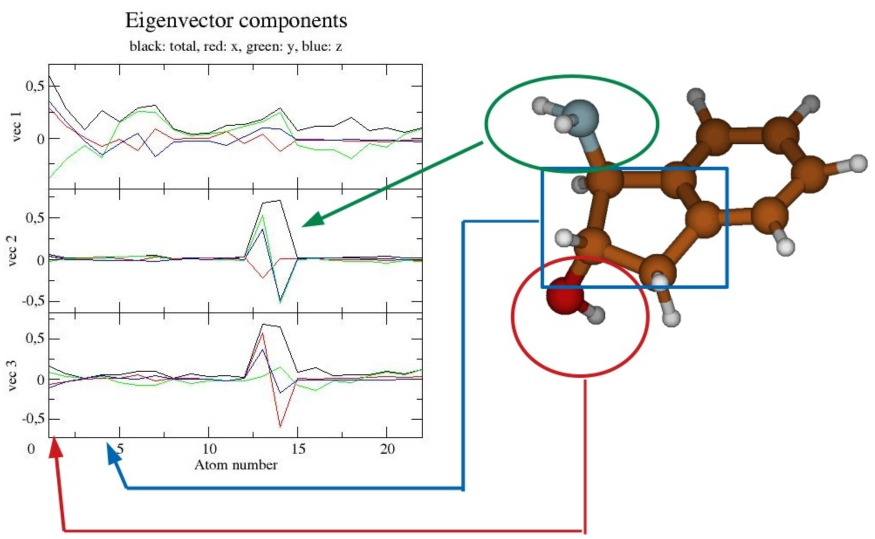

Cartesian coordinates, frequencies and Rotational strengths of the constrained-optimized clusters of (1S,2S)-trans-1-amino-2-indanol with 3 molecules of dimethyl sulfoxide from the rEMCS analysis carried out onto the *MD-ellipsoid* trajectories in turn obtained from EMCS applied to the initial *MD-constrained* trajectories. The structures are also depicted in Figure S7. Frequencies are reported in cm^{-1}, Rotational strengths are reported in 10^{-44} esu^2cm^2

$1A_{up}$

6	-1.123687	-3.995083	-0.798175
6	-0.810610	-2.659409	-0.542214
6	-0.227392	-2.279906	0.671137
6	0.059606	-3.243321	1.640967
6	-0.224374	-4.586605	1.379537
6	-0.839489	-4.957173	0.177235
6	-0.327177	-0.774265	0.868703
6	-0.434515	-0.227904	-0.608314
6	-0.702985	-1.489580	-1.505545
8	0.738002	0.448013	-1.007528
7	-1.476968	-0.487470	1.753286
8	2.700321	-0.992233	-1.176635
16	3.291778	-1.817697	-0.033025
6	5.097031	-1.539862	-0.114206
6	3.270324	-3.561382	-0.578948
6	1.894581	3.161196	-0.053083
16	3.118326	4.518284	-0.001019
6	1.917295	5.885870	-0.262455
8	3.641093	4.645481	1.415779
8	-3.979634	0.702433	0.153037
16	-5.152047	1.316746	-0.581907
6	-5.400250	3.004754	0.094988
6	-6.678678	0.583539	0.123647
1	1.448505	-0.203827	-1.155704
1	-1.263816	0.483632	-0.676305
1	0.147752	-1.636325	-2.181359
1	-1.589816	-1.360203	-2.139685
1	0.573752	-0.346833	1.321760
1	-2.269742	-0.169940	1.191719
1	-1.239878	0.324891	2.322350
1	0.468515	-2.947422	2.604458
1	0.000463	-5.344984	2.125847
1	-1.085282	-6.000960	-0.004327
1	-1.566901	-4.289541	-1.747257
1	2.223368	-3.874303	-0.595719
1	3.704062	-3.624389	-1.580595
1	3.825486	-4.179457	0.133187
1	5.602650	-2.175577	0.619065
1	5.448007	-1.750861	-1.127737
1	5.269851	-0.488062	0.124972
1	-7.560195	1.071638	-0.303246
1	-6.675557	-0.476150	-0.142306

1	-6.659698	0.687792	1.212216
1	-4.532980	3.599535	-0.202206
1	-6.310756	3.445873	-0.322029
1	-5.454474	2.950021	1.185795
1	2.423229	2.233866	0.173844
1	1.101822	3.315695	0.685555
1	1.478013	3.067366	-1.058508
1	2.479618	6.823081	-0.262667
1	1.405847	5.757615	-1.221008
1	1.201665	5.888027	0.565217

Frequencies

1004.46759	-68.2965012
1012.96362	110.882896
1039.04346	-23.3901997
1082.84851	3.47539997
1085.34204	7.81540012
1112.62817	5.90850019
1170.77234	-11.2872000
1176.59875	0.488799989
1185.35254	-44.0656013
1199.49304	-20.7112007
1217.71960	-4.95819998
1230.32959	12.9799995
1275.16077	9.25940037
1309.00171	-0.121200003
1332.23914	0.243499994
1341.42090	3.38870001
1357.06628	-5.14690018
1409.26636	55.8601990
1459.49048	23.9773006
1479.92566	-13.0386000
1483.39673	30.2334003
1501.86084	4.38749981
1612.88550	3.39650011
1627.28064	2.94479990
1682.96326	18.1240005
3005.20264	24.5323009
3032.02222	21.5608997
3033.72729	-12.0429001
3047.29956	-0.549899995
3126.29614	-0.765200019
3134.53491	3.77080011
3144.67578	0.644299984
3155.63525	-6.30840015
3435.91406	-27.3169994
3487.30029	82.7389984
3499.31738	19.4459991

$2A_{up}$

6	-1.50242	-2.79476	-0.08548
6	-0.87229	-1.56139	-0.25949
6	-0.33991	-0.86548	0.83009

6	-0.44593	-1.39993	2.11708
6	-1.06007	-2.64442	2.29771
6	-1.608	-3.3273	1.20414
6	-0.04767	0.57945	0.45566
6	0.15366	0.50647	-1.10543
6	-0.34938	-0.91721	-1.53232
8	1.4919	0.77991	-1.4951
7	-1.13746	1.45868	0.92041
8	2.84662	-1.09357	-0.74698
16	2.86161	-2.62311	-0.73226
6	2.84634	-3.11539	1.02459
6	4.5876	-3.10083	-1.10175
8	1.01466	3.48781	1.47819
16	2.16969	4.10304	0.70154
6	1.72766	4.10703	-1.07829
6	3.48098	2.82812	0.56445
8	-3.31111	0.92935	-1.3594
16	-4.54368	0.91284	-0.46661
6	-4.14781	-0.06503	1.02915
6	-5.71128	-0.29238	-1.20861
1	2.03031	-0.00494	-1.28218
1	-0.46645	1.27245	-1.57982
1	0.4759	-1.50344	-1.95041
1	-1.11456	-0.84198	-2.31453
1	0.87856	0.94142	0.90912
1	-1.72422	1.69272	0.11836
1	-0.69427	2.33084	1.2152
1	-0.08473	-0.83707	2.97514
1	-1.14149	-3.07083	3.29504
1	-2.10278	-4.28378	1.35709
1	-1.89243	-3.34441	-0.94005
1	4.78528	-2.81132	-2.13636
1	5.26245	-2.562	-0.43097
1	4.70287	-4.18306	-0.98965
1	1.87361	-2.82117	1.42606
1	2.97174	-4.19993	1.09761
1	3.64606	-2.58954	1.55381
1	-6.56496	-0.44135	-0.54027
1	-6.05137	0.12388	-2.15993
1	-5.18487	-1.23504	-1.3844
1	-3.29359	0.4318	1.50139
1	-5.02017	-0.06967	1.69087
1	-3.857	-1.08154	0.74997
1	3.81834	2.60694	1.58018
1	3.06702	1.93479	0.08825
1	4.31279	3.23334	-0.021
1	2.53859	4.58604	-1.63714
1	1.56841	3.08058	-1.423
1	0.81572	4.70138	-1.17656

Frequencies

1008.52289	177.595993
1026.80872	43.1324005

1065.62537	-31.3586998
1074.42761	-40.5049019
1080.28564	1.85459995
1095.55554	-9.64509964
1138.73547	-10.8424997
1170.57214	-2.80850005
1187.13770	-29.4890995
1200.38782	5.07999994E-02
1217.79370	-35.0172997
1246.84143	24.9815006
1278.40515	5.10750008
1286.04663	7.64020014
1334.46118	-3.89269996
1345.51465	2.63590002
1356.97705	-3.43980002
1412.27148	37.0469017
1465.16089	9.03699970
1472.27319	19.6476002
1475.34534	-32.8605003
1484.78198	9.50179958
1606.71790	3.11080003
1616.68518	6.32560015
1677.13281	-16.7714005
2315.02954	-2.19409990
3019.38574	14.8031998
3044.49634	-22.0709000
3057.45630	9.36170006
3064.45215	7.51210022
3127.55762	1.37740004
3140.22729	1.64619994
3152.26001	-3.44440007
3421.24121	97.6197968
3487.11548	-4.59679985
3501.27759	-40.3237000

$1B_{up}$

6	2.8192	-4.10654	-0.35135
6	2.14314	-2.90414	-0.57798
6	0.76257	-2.83148	-0.36132
6	0.02119	-3.98512	-0.09583
6	0.68763	-5.2067	0.02123
6	2.08279	-5.26415	-0.08873
6	0.2668	-1.40111	-0.3609
6	1.53918	-0.53437	-0.60137
6	2.75079	-1.51494	-0.63247
8	1.63329	0.47027	0.39856
7	-0.83834	-1.08563	-1.28538
8	3.39773	1.80428	-0.63137
16	4.4247	2.45415	0.30557
6	4.72981	1.2446	1.6463
6	3.50203	3.67917	1.30295

8	-3.67223	-1.82587	0.72051
16	-4.84678	-0.97991	1.17799
6	-5.42196	-0.00956	-0.26714
6	-4.17061	0.44693	2.10994
6	-0.62837	3.00054	0.09957
16	-2.24624	2.75836	-0.72192
8	-2.86868	4.12517	-0.93331
6	-1.57995	2.16139	-2.31734
1	2.29879	1.10359	0.04628
1	1.43422	-0.06139	-1.58748
1	3.37369	-1.34488	0.25475
1	3.39167	-1.35223	-1.50835
1	-0.08747	-1.14629	0.64598
1	-1.72767	-1.14337	-0.78781
1	-0.87241	-1.76322	-2.04461
1	-1.05461	-3.92292	0.05539
1	0.12541	-6.11235	0.23494
1	2.5961	-6.21196	0.05426
1	3.9045	-4.15181	-0.41698
1	4.17684	4.13821	2.03142
1	3.13407	4.43822	0.60889
1	2.65906	3.19204	1.8009
1	3.76892	0.84497	1.98252
1	5.3333	0.43967	1.22074
1	5.27639	1.72983	2.45987
1	-3.43923	0.97496	1.49108
1	-4.98755	1.11603	2.3974
1	-3.68817	0.04487	3.00412
1	-6.21612	0.67667	0.04232
1	-4.57971	0.54371	-0.69233
1	-5.81166	-0.72195	-0.99827
1	-2.42602	2.10631	-3.00796
1	-0.84165	2.87152	-2.70246
1	-1.14905	1.16379	-2.16162
1	-0.07739	2.05462	0.16299
1	-0.06109	3.74795	-0.46479
1	-0.83803	3.38516	1.10169

Frequencies

1011.95770	22.1581001
1026.98157	-42.4427986
1075.55212	-17.8477993
1083.26025	9.09280014
1103.64294	-17.2015991
1139.65234	1.89800000
1180.30676	-4.81820011
1191.87524	4.39480019
1206.23486	0.736199975
1221.35168	-12.1314001
1237.09802	-13.1992998
1272.52991	7.91270018
1296.09448	-17.0044994
1312.60229	16.3118992

1343.15393	-2.07800007
1387.26917	4.07959986
1416.43567	52.0745010
1468.86975	0.597100019
1476.29895	-3.98569989
1486.55396	1.86099994
1511.32935	19.6079998
1606.96985	2.07960010
1625.72217	2.25710011
1676.38416	-22.6488991
2316.14941	-3.16269994
2987.16870	-14.7990999
3004.79492	8.46949959
3015.02832	4.61800003
3034.58643	-15.9567003
3132.61304	-0.00000000
3142.56177	-0.748899996
3156.96997	-0.978200018
3319.83521	-319.616394
3440.07617	0.505800009
3524.59424	-0.523899972

$2B_{up}$

6	-0.07043	-4.4648	-0.49767
6	-0.49417	-3.22892	-0.00125
6	0.3943	-2.14541	0.02832
6	1.74851	-2.33061	-0.26658
6	2.18871	-3.59717	-0.66054
6	1.2818	-4.65567	-0.79127
6	-0.32333	-0.83597	0.30538
6	-1.81891	-1.23511	0.44118
6	-1.93	-2.75549	0.13825
8	-2.59924	-0.41541	-0.4174
7	0.1509	0.00037	1.41363
8	-5.17289	-0.40472	0.25478
16	-5.44141	1.00635	0.78808
6	-4.81263	2.16501	-0.48284
6	-4.16289	1.35909	2.05162
8	1.04228	2.22844	-0.2146
16	1.12893	2.47548	-1.72052
6	-0.57882	2.33319	-2.36936
6	1.81808	0.97459	-2.50285
8	4.21136	0.42031	-0.28513
16	4.10456	0.80463	1.1835
6	3.94675	2.62548	1.26785
6	5.8043	0.69951	1.8652
1	-3.54586	-0.49574	-0.15013
1	-2.10307	-1.05345	1.4883
1	-2.4719	-2.89525	-0.80557
1	-2.49207	-3.29436	0.91222
1	-0.26593	-0.20727	-0.58995
1	0.79533	0.70049	1.05379

1	0.64424	-0.57074	2.09776
1	2.4486	-1.4978	-0.23459
1	3.23772	-3.75166	-0.9017
1	1.62801	-5.62354	-1.14619
1	-0.77862	-5.28176	-0.6211
1	-4.34973	0.68363	2.88985
1	-3.16996	1.17167	1.63206
1	-4.26349	2.39647	2.38495
1	-5.44783	2.04732	-1.3639
1	-4.87871	3.19121	-0.10923
1	-3.7813	1.89094	-0.72186
1	1.93118	1.15927	-3.57593
1	2.78958	0.80392	-2.02961
1	1.1706	0.11228	-2.32377
1	-1.12841	3.20905	-2.01522
1	-0.55268	2.33206	-3.46342
1	-1.04941	1.42349	-1.98324
1	6.0956	-0.35324	1.83989
1	6.48362	1.28439	1.23827
1	5.81393	1.06288	2.89735
1	4.73885	3.08537	0.66876
1	2.96201	2.86079	0.85395
1	4.01038	2.94836	2.31186

Frequencies

1007.64258	-4.97510004
1017.58478	113.456100
1019.30841	-36.9497986
1037.61475	-50.7610016
1076.12146	-54.2314987
1078.02966	13.4256001
1087.09314	-0.459500015
1103.67725	-9.32890034
1145.40015	-2.99950004
1181.03784	-0.160699993
1193.00269	8.37950039
1206.12402	1.96120000
1221.05017	-2.87369990
1239.40698	-4.89400005
1272.70715	7.25479984
1295.97754	-16.7359009
1310.44897	12.2240000
1342.99011	2.49880004
1380.64868	-2.87599993
1403.88184	38.8890991
1467.56274	1.71560001
1475.22559	5.56559992
1484.88708	-27.0426006
1491.96985	43.2713013
1604.45361	5.57910013
1623.98315	0.367500007
1650.83850	20.5823002
2316.92261	-1.12559998

2969.45654	7.54120016
3003.30933	19.7087002
3034.46753	-1.08959997
3037.36938	-4.16830015
3130.35083	1.52980006
3139.82495	4.28620005
3154.01489	-4.77640009
3345.71777	303.800903
3455.57520	4.66029978
3541.09570	24.1247005

1A$_{down}$

6	-1.23334	-4.6141	0.9032
6	-1.06853	-3.22713	0.89378
6	0.10556	-2.65967	0.38721
6	1.15236	-3.46895	-0.06255
6	0.99527	-4.85625	-0.02889
6	-0.18859	-5.42617	0.45615
6	0.10211	-1.15023	0.54129
6	-1.36514	-0.80001	0.93201
6	-2.11549	-2.15614	1.12415
8	-1.93569	0.01651	-0.08677
7	1.08013	-0.61328	1.50704
8	-4.18418	1.22534	0.4062
16	-4.84018	1.36256	-0.97061
6	-4.8226	-0.30258	-1.73192
6	-3.58934	2.11859	-2.07337
8	3.44289	-0.89817	-0.47947
16	4.02699	0.0818	-1.48834
6	5.75075	-0.45857	-1.79636
6	4.44353	1.613	-0.57907
6	-0.44745	3.20987	0.22893
16	1.25178	3.49024	0.85268
8	1.43517	4.98466	1.03076
6	0.99508	2.72803	2.49537
1	-2.73208	0.47196	0.28154
1	-1.34479	-0.24309	1.87774
1	-2.91573	-2.23355	0.37794
1	-2.59563	-2.22708	2.10944
1	0.31391	-0.65385	-0.41198
1	1.9995	-0.62526	1.05923
1	1.14076	-1.24108	2.3095
1	2.07147	-3.01717	-0.4283
1	1.7971	-5.50052	-0.38171
1	-0.30026	-6.50768	0.47181
1	-2.16015	-5.0602	1.25836
1	-3.42403	3.13712	-1.71435
1	-2.66264	1.54204	-2.00672
1	-3.97386	2.14405	-3.09732
1	-3.80862	-0.70454	-1.65722
1	-5.51791	-0.92296	-1.16134
1	-5.15557	-0.2324	-2.77164

1	4.96989	2.30178	-1.24703
1	3.50362	2.06508	-0.2504
1	5.06074	1.36466	0.28956
1	5.69984	-1.41577	-2.32053
1	6.26426	0.27963	-2.41961
1	6.26459	-0.58903	-0.8396
1	0.87997	1.64486	2.35775
1	1.89244	2.94265	3.08222
1	0.12459	3.17653	2.98395
1	-1.14838	3.75462	0.86934
1	-0.48546	3.62219	-0.78318
1	-0.68652	2.14156	0.20819

Frequencies

1001.27399	-48.7653008
1011.55139	35.0948982
1012.74750	-21.0825005
1024.55286	13.7157001
1068.83447	-15.1043997
1082.22559	8.96570015
1117.08130	-29.9796009
1127.80847	31.5109005
1182.75891	-2.32750010
1192.01868	4.19759989
1206.61597	1.78330004
1229.67969	-7.50790024
1231.50964	-5.50820017
1278.99548	6.18060017
1293.01855	2.11069989
1319.73975	-20.4962006
1345.85938	1.61080003
1375.44116	-28.0158005
1416.45325	74.4993973
1471.93689	-3.49160004
1475.99255	10.4609003
1485.74231	-14.9554005
1514.98926	51.2986984
1608.87451	5.03789997
1626.17053	8.42529964
1675.96387	-25.1166992
2316.85425	-2.99320006
2994.10645	7.85529995
3005.95679	-7.90700006
3033.61230	1.09809995
3037.58301	-5.36829996
3133.04810	0.112499997
3147.73853	3.67109990
3157.26660	-6.63490009
3311.79810	-71.4629974
3413.90210	30.9473000
3492.62720	32.2635994

2A$_{down}$

6	2.4878	1.85022	2.38201
6	1.4019	1.2208	1.76645
6	1.45088	-0.15037	1.48582
6	2.59612	-0.89399	1.77947
6	3.69012	-0.25991	2.3765
6	3.63888	1.10895	2.66827
6	0.20251	-0.61641	0.75524
6	-0.78536	0.57658	0.88502
6	-0.00687	1.74577	1.57072
8	-1.93898	0.16744	1.58913
7	0.36823	-1.04055	-0.63843
8	-3.47044	2.02213	0.90742
16	-4.45294	1.79806	-0.24569
6	-3.46391	1.72159	-1.78195
6	-5.22774	3.42463	-0.55058
8	2.06006	0.84099	-2.48774
16	3.5474	1.14631	-2.53608
6	4.42409	-0.22627	-1.69607
6	3.89209	2.42074	-1.26512
8	0.1334	-4.0268	0.08277
16	-1.30861	-4.35759	-0.27581
6	-1.74318	-3.38058	-1.7661
6	-2.42174	-3.47099	0.88461
1	-2.64944	0.80784	1.38669
1	-1.05395	0.88469	-0.13592
1	-0.47282	1.95687	2.54127
1	-0.04825	2.67389	0.98586
1	-0.23311	-1.46921	1.28473
1	0.78282	-1.97234	-0.65223
1	0.96955	-0.39819	-1.16109
1	2.62697	-1.9587	1.556
1	4.5808	-0.83209	2.62552
1	4.48957	1.59242	3.14289
1	2.43942	2.9053	2.64506
1	-4.44449	4.17789	-0.67197
1	-5.86271	3.36954	-1.43969
1	-5.83433	3.65967	0.3268
1	-2.85006	0.81922	-1.72741
1	-4.13716	1.66519	-2.64275
1	-2.81583	2.60004	-1.84654
1	-3.45708	-3.71262	0.62135
1	-2.2037	-3.85842	1.88319
1	-2.25603	-2.38923	0.86797
1	-1.21797	-3.83271	-2.61178
1	-2.82364	-3.43198	-1.93419
1	-1.39138	-2.35286	-1.6134
1	3.38941	3.33532	-1.58963
1	3.49524	2.10237	-0.29686
1	4.97129	2.59457	-1.20844
1	4.26244	-1.12309	-2.2992
1	5.49395	0.00036	-1.65149
1	4.01361	-0.37302	-0.69279

Frequencies

1010.32440	229.698700
1014.30670	-120.357803
1026.61401	-58.1025009
1027.62854	20.0578003
1080.83691	-14.5436001
1089.22119	-16.2803001
1093.17834	-34.1017990
1114.51074	-21.2313004
1138.29395	9.53670025
1180.72266	-2.99690008
1199.22266	6.17980003
1205.22095	-0.722599983
1232.78699	-8.70699978
1244.83154	1.64499998
1284.79688	0.633199990
1298.67993	14.8094997
1324.38367	-39.9244995
1347.89526	7.62419987
1377.17395	29.0261002
1429.76465	-4.81559992
1469.74731	-1.08249998
1477.00366	23.6026001
1482.29968	-31.7220993
1491.99097	47.9323997
1607.78833	4.14720011
1625.21863	8.30510044
1717.84363	-78.5710983
2320.25684	1.05100000
2982.73340	-6.19350004
3010.00122	16.7329998
3043.16138	-8.72070026
3062.13037	5.68190002
3136.27563	-4.14000005E-02
3147.56348	-7.16450024
3161.57690	3.72919989
3399.97925	123.049500
3466.21899	176.718704
3497.35327	-112.314697

$1B_{down}$

6	-2.82939	-0.74703	2.45724
6	-1.71876	-0.52014	1.64573
6	-1.54232	0.7176	1.01338
6	-2.48697	1.73215	1.16618
6	-3.61398	1.49911	1.96228
6	-3.77225	0.27456	2.62201
6	-0.1366	0.84726	0.46597
6	0.46903	-0.5952	0.59275
6	-0.69061	-1.50143	1.10365
8	1.57008	-0.56201	1.47462
7	-0.06645	1.51171	-0.84028

8	3.21048	-2.23551	0.66292
16	4.51877	-1.83498	-0.02577
6	4.15718	-1.66229	-1.80389
6	5.49884	-3.37368	-0.11289
8	3.23347	1.45051	-1.65686
16	3.82459	2.63003	-0.88968
6	5.4387	2.07782	-0.21927
6	2.94712	2.72317	0.71098
6	-4.58233	-1.29194	-1.26267
16	-6.19778	-0.62659	-1.82831
8	-7.26789	-1.1792	-0.91266
6	-5.86961	1.10914	-1.32529
1	2.23504	-1.22217	1.18797
1	0.79692	-0.93138	-0.4033
1	-0.31033	-2.21225	1.84568
1	-1.13991	-2.08597	0.28901
1	0.43416	1.46664	1.16789
1	-0.63797	1.0002	-1.5129
1	0.89238	1.46739	-1.19305
1	-2.33142	2.69577	0.68747
1	-4.35599	2.28284	2.09584
1	-4.63903	0.11194	3.25759
1	-2.97381	-1.70869	2.94448
1	6.42207	-3.19111	-0.67059
1	5.7335	-3.66264	0.91398
1	4.90041	-4.15486	-0.58945
1	3.63053	-0.71069	-1.92438
1	5.09855	-1.63592	-2.3619
1	3.53901	-2.50731	-2.12048
1	-5.06512	1.52372	-1.9408
1	-6.79292	1.66817	-1.49639
1	-5.59957	1.14648	-0.26627
1	-4.47184	-1.12518	-0.18732
1	-4.58666	-2.36425	-1.47389
1	-3.76959	-0.80813	-1.81315
1	1.9579	3.13853	0.50337
1	2.83665	1.71361	1.12257
1	3.49172	3.38458	1.39164
1	5.30554	1.1551	0.3528
1	6.09597	1.89503	-1.07309
1	5.86216	2.86829	0.40754

Frequencies

1001.68829	-1.63639998
1038.87488	-9.06869984
1073.48083	-33.9790993
1074.86023	29.1287994
1075.44446	-40.7186012
1080.68762	34.8605995
1088.02087	-48.7995987
1134.98328	-47.9085007
1173.08911	-8.50669956

1185.27246	15.6629000
1205.52844	-2.74559999
1213.95007	-3.72839999
1239.76355	-0.241099998
1273.54773	0.112999998
1295.38416	15.4252996
1309.32056	-39.7246017
1345.75940	3.08159995
1359.70923	20.5643997
1422.91187	26.2765007
1456.20789	7.58479977
1466.23047	18.5709000
1475.45544	6.66459990
1483.05627	0.454499990
1607.23071	-1.38300002
1624.44385	-18.2318993
1679.67725	10.0361996
2322.57520	-3.50000001E-02
2959.81104	-18.1644993
2996.43896	2.05279994
3021.30396	3.13059998
3051.35254	-9.58590031
3133.86694	0.415600002
3149.78296	0.710600019
3158.59106	3.58130002
3414.68799	-496.245514
3418.50488	812.908813
3496.16235	-5.03299999

2B$_{down}$

6	3.6237	-2.62361	1.75104
6	2.51532	-1.90499	1.30669
6	2.21236	-1.83902	-0.05962
6	3.03723	-2.45912	-0.99655
6	4.163	-3.16146	-0.5552
6	4.43981	-3.26424	0.81241
6	0.81257	-1.29644	-0.28519
6	0.38546	-0.76687	1.12416
6	1.61993	-0.92857	2.05691
8	-0.78059	-1.41097	1.58818
7	0.67703	-0.33524	-1.39147
8	-2.24357	0.33424	2.61967
16	-2.64018	1.74898	2.18505
6	-2.32911	1.95754	0.39296
6	-1.31913	2.87468	2.76406
8	-2.68749	-0.43161	-1.87177
16	-3.05362	-1.91123	-1.79253
6	-4.76316	-2.05515	-2.43921
6	-3.43494	-2.29556	-0.04988
6	2.0076	2.52303	-2.79087
16	2.29529	3.07446	-1.06794
6	2.131	4.87213	-1.38511

8	1.07332	2.66396	-0.25131
1	-1.30965	-0.73959	2.07365
1	0.17629	0.30704	1.01761
1	1.30385	-1.27564	3.04745
1	2.14776	0.02502	2.18981
1	0.16607	-2.15182	-0.52032
1	0.83395	0.59917	-1.01209
1	-0.2987	-0.33139	-1.69825
1	2.79221	-2.40423	-2.05451
1	4.8155	-3.6496	-1.27547
1	5.30417	-3.83306	1.1471
1	3.86596	-2.67068	2.8107
1	-1.29071	2.80712	3.8541
1	-0.35862	2.57831	2.33342
1	-1.57033	3.89582	2.46082
1	-2.78718	1.12259	-0.14609
1	-2.80578	2.89806	0.09565
1	-1.25904	1.99503	0.1709
1	2.85031	2.83611	-3.41506
1	1.93517	1.43116	-2.7659
1	1.06839	2.94571	-3.15979
1	1.1442	5.07576	-1.81081
1	2.23243	5.38259	-0.4244
1	2.92407	5.19934	-2.06422
1	-4.23482	-1.64023	0.30893
1	-3.73206	-3.34652	0.02647
1	-2.51667	-2.11137	0.52068
1	-5.40323	-1.33225	-1.9249
1	-4.72949	-1.82506	-3.50678
1	-5.13007	-3.07501	-2.28925

Frequencies

1003.08063	-41.6596985
1004.32520	527.356506
1009.47357	-332.162689
1012.85461	-101.008202
1046.09070	31.5552998
1074.65125	7.89680004
1079.23743	-6.68909979
1089.32751	-56.2793999
1097.35730	-3.96169996
1147.34546	-49.8997993
1175.75647	-7.79519987
1183.65479	14.3034000
1210.83191	-5.60260010
1238.64050	-10.2420998
1242.73621	-15.2109003
1281.84045	-11.3269997
1287.29871	31.7889004
1340.26062	-20.2353992
1348.57141	-9.80169964
1388.71033	-7.07089996
1431.03687	43.0731010

1462.22815	-5.69969988
1472.80676	7.96029997
1482.55188	-14.5085001
1503.39648	46.0438995
1608.70020	-2.12719989
1626.38306	-11.2796001
1702.25049	3.74480009
2318.29150	1.43340003
2977.62158	-13.1148996
3002.43506	21.2980995
3007.10913	11.4413004
3047.18530	-11.8162003
3130.01978	-0.198799998
3144.64551	1.72019994
3155.58618	-4.36259985
3379.93750	44.5624008
3417.36279	137.320297
3479.52222	-61.7350998

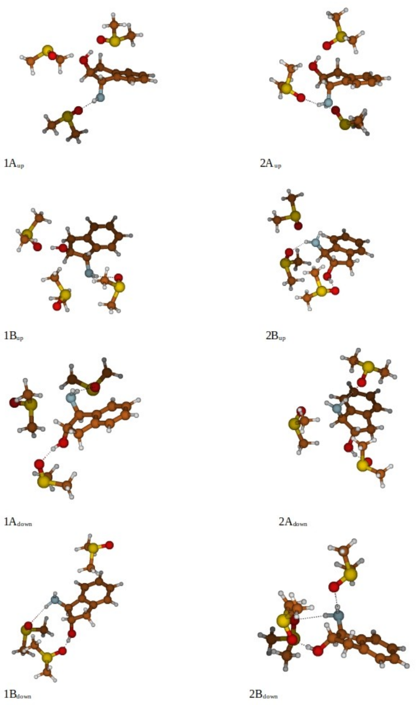

Figure S7. Pictorial representation of the representative conformations of (1S,2S)-trans-1-amino-2-indanol with 3 molecules of dimethyl sulfoxide from rEMCS analysis

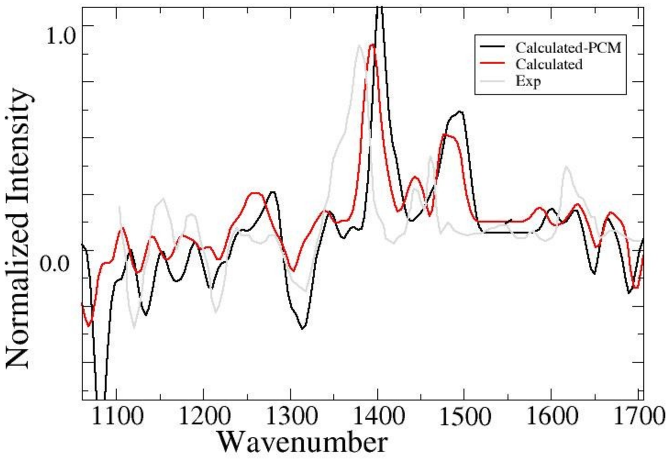

Figure S8. Superposition of the Calculated spectra without (red) and with (black) the PCM and the experimental one (grey).

S4. Supplementary results for Di-alanine – B3LYP/6-31+G*

Cartesian coordinates of the constrained-optimized ADP$(H_2O)_8$ clusters from rEMCS analysis carried out onto the *MD-ellipsoid* trajectories in turn obtained from EMCS applied to the initial *MD-constrained* trajectories.

- PII conformation The structures are also depicted in Figure S8.

A

6	-2.76153	1.46185	-0.99844
6	-1.36733	1.22537	-0.46552
7	-1.18909	0.20617	0.40872
6	0.18052	-0.11111	0.81607
6	0.82038	-0.9069	-0.32987
7	2.07436	-0.60189	-0.65104
6	2.84257	-1.24067	-1.72046
8	-0.40715	1.92146	-0.83564
6	0.195	-0.93117	2.10967
8	0.16436	-1.9152	-0.71132
8	-2.16417	-1.91187	-2.13867
8	0.44708	4.23791	-2.04463
8	-0.15508	4.63051	0.69523
8	3.73972	1.15185	0.99463
8	3.67132	-0.87251	2.73988
8	-3.37784	-1.96082	0.53679
8	-4.09905	-0.55272	2.84701
8	1.37771	-4.02201	-1.6328
1	-3.07944	2.46956	-0.70925
1	-3.49192	0.73399	-0.63729
1	-2.7313	1.4149	-2.09155
1	-1.94025	-0.44171	0.63441
1	0.69796	0.83901	0.96355
1	-0.31014	-1.89223	1.96807
1	1.22661	-1.11984	2.42414
1	-0.32511	-0.37645	2.89765
1	2.50071	0.18472	-0.17139
1	3.75867	-0.66356	-1.86365
1	2.26923	-1.23371	-2.65251
1	3.08719	-2.27609	-1.47631
1	0.08635	3.34794	-1.81612
1	-0.13439	4.59633	-2.73113
1	0.10592	4.64211	-0.24987
1	-0.1335	5.56606	0.942
1	-3.16594	-2.06391	-0.42207
1	-3.07715	-2.78101	0.95623
1	-4.01831	-0.90718	1.94211
1	-4.8485	0.05953	2.83847
1	-1.2915	-1.88116	-1.68062
1	-2.08724	-2.64555	-2.76678
1	1.15524	-4.7761	-1.06831
1	0.86309	-3.25697	-1.29391
1	4.57766	1.37851	0.55876

```
1    3.30521   2.00342   1.17903
1    3.93891  -0.76066   3.66349
1    3.83211  -0.02706   2.28661

B
6   -1.22817  -3.02169   1.4571
6   -0.25836  -2.0754    0.7793
7   -0.73597  -1.44882  -0.31229
6   -0.07924  -0.39526  -1.08856
6   -0.20588   0.95289  -0.36207
7    0.88646   1.72138  -0.35189
6    0.96856   3.03069   0.29838
8    0.9485   -1.99901   1.12057
6   -0.72343  -0.30667  -2.47524
8   -1.34717   1.26576   0.02264
8    1.85992  -0.54625   2.65315
8   -3.98338   0.42781   0.23404
8    0.48484   1.86499   3.14976
8    2.8736   -2.92562  -0.966
8    3.19193  -0.20342  -1.82138
8    5.06098   2.21031  -0.78337
8   -4.20283   3.66959  -0.62047
8   -3.93756  -2.50603  -0.96705
1   -1.25196  -3.95578   0.88234
1   -2.24618  -2.62719   1.48106
1   -0.88235  -3.24891   2.4673
1   -1.74234  -1.53622  -0.45481
1    0.97315  -0.65861  -1.20636
1   -1.77729  -0.01701  -2.40058
1   -0.21309   0.44791  -3.0837
1   -0.65027  -1.27218  -2.98616
1    1.76374   1.27092  -0.59963
1    2.00468   3.19715   0.59651
1    0.34465   3.02901   1.18903
1    0.65931   3.8187   -0.39897
1    2.28893  -1.05758   3.354
1    1.5032   -1.20247   2.03257
1    2.25414  -2.57648  -0.2862
1    3.49697  -3.44032  -0.42783
1   -3.07225   0.73211   0.02896
1   -4.1015    0.70015   1.15665
1   -4.38347  -1.68428  -0.68531
1   -3.95369  -2.4984   -1.9356
1    0.99616   1.02609   3.08343
1   -0.41572   1.55951   3.33289
1   -3.99041   3.33114  -1.50259
1   -3.59131   3.20339  -0.02965
1    3.08146  -0.44351  -2.75499
1    3.44122  -1.05768  -1.40799
1    4.63084   1.35571  -0.96942
1    5.34837   2.52439  -1.65432
```

C

6	2.18678	2.58196	-0.10932
6	0.82856	1.92858	-0.24613
7	0.71487	0.99554	-1.2098
6	-0.47026	0.17795	-1.39481
6	-0.53265	-0.97162	-0.36571
7	-1.73722	-1.19763	0.16572
6	-2.0304	-2.22302	1.16746
8	-0.15004	2.23304	0.47238
6	-0.48248	-0.39636	-2.82029
8	0.5135	-1.57774	-0.05538
8	1.1159	-3.88923	1.01735
8	-0.19382	4.5446	1.78958
8	-0.43847	0.83707	2.87749
8	1.22572	-1.7422	2.71789
8	-4.90831	-1.33097	-0.87783
8	-4.18433	1.35268	-1.42556
8	4.22042	-0.24124	-2.6267
8	4.29539	-0.88903	0.10781
1	2.21981	3.17229	0.80705
1	2.36859	3.2536	-0.95715
1	2.97964	1.82529	-0.08686
1	1.57171	0.67964	-1.65372
1	-1.33561	0.83189	-1.25681
1	0.34354	-1.10283	-2.96185
1	-1.41999	-0.92931	-3.00713
1	-0.38749	0.41039	-3.55519
1	-2.53415	-0.69034	-0.20223
1	-2.98316	-2.6916	0.91025
1	-2.09606	-1.76488	2.16051
1	-1.23656	-2.97145	1.17431
1	-0.3959	1.38214	2.06739
1	-0.94796	1.38142	3.49374
1	-0.16434	3.68391	1.32357
1	0.2181	4.40318	2.65381
1	4.43525	-0.96065	-3.2409
1	5.08049	0.13761	-2.38262
1	3.65502	-1.42902	0.59919
1	4.04472	-0.95967	-0.83007
1	1.26171	-3.53631	1.91109
1	0.90728	-3.10973	0.4806
1	0.95447	-1.44805	1.82935
1	0.89827	-1.03228	3.29584
1	-4.92789	-0.36984	-1.05051
1	-5.79315	-1.56904	-0.56534
1	-4.38549	1.80646	-2.25806
1	-4.13935	2.05099	-0.75332

D

6	3.31848	1.17262	-0.03323
6	1.82005	1.20197	-0.23667
7	1.24211	0.09591	-0.75431

6	-0.19563	-0.06879	-0.88188
6	-0.78358	-0.43774	0.49724
7	-1.92353	0.15713	0.848
6	-2.58576	-0.08475	2.12596
8	1.14492	2.19856	0.07659
6	-0.5155	-1.1381	-1.93527
8	-0.09261	-1.12782	1.27869
8	-0.21772	3.1789	2.16064
8	0.59097	-3.87656	0.96867
8	0.14556	-0.93694	4.17168
8	-3.63788	1.15903	-1.36914
8	-5.53556	-1.013	-2.19446
8	2.80922	-2.65005	-0.32666
8	3.43368	-2.09036	-2.81192
8	1.64329	5.02617	-1.41353
1	3.78154	1.94083	-0.66233
1	3.76514	0.19939	-0.2542
1	3.52827	1.43202	1.00922
1	1.81096	-0.74015	-0.82883
1	-0.61096	0.89141	-1.19818
1	-0.12804	-2.11923	-1.63778
1	-1.59868	-1.21755	-2.07147
1	-0.06122	-0.87051	-2.89427
1	-2.42672	0.69925	0.14512
1	-3.52104	0.4775	2.13401
1	-1.95987	0.25361	2.95623
1	-2.79872	-1.15037	2.26469
1	0.27935	2.82783	1.39939
1	0.05277	2.64086	2.91887
1	1.43567	5.69287	-0.74166
1	1.49134	4.17162	-0.96775
1	2.00479	-3.10441	0.0249
1	3.49787	-3.33323	-0.28602
1	3.80467	-1.1966	-2.83327
1	3.12446	-2.24228	-1.89864
1	-0.14545	-4.38064	0.59339
1	0.22701	-3.0013	1.23043
1	0.83577	-1.56725	4.4246
1	0.0627	-1.01604	3.19912
1	-4.31356	0.50843	-1.64935
1	-3.95843	2.02868	-1.65054
1	-5.62742	-1.23677	-3.13345
1	-6.40161	-1.17408	-1.78951

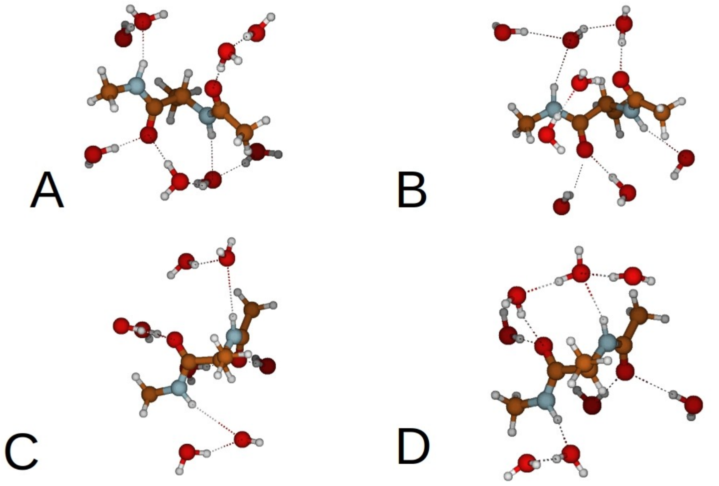

Figure S8. Pictorial representation of the representative conformations of ADP(H_2O)$_8$ free energy basins for the MD-constrained simulation of ADP in the PII conformation.

Below we report the VCD signals of the structures previously reported calculated at the same level of theory. Frequencies are reported in cm^{-1}, Rotational strengths are reported in 10^{-44} esu^{2}cm^2

A

689.325195	-98.9399033
728.255493	-142.281097
795.482727	-47.6843987
884.808899	10.1567001
945.057007	5.63259983
973.770874	-2.78469992
1035.13196	6.27360010
1048.18604	6.10109997
1072.67139	-19.3234997
1078.62048	-6.33050013
1131.21997	39.9544983
1152.42200	-9.99790001
1196.33875	-90.7856979
1202.50732	41.9674988
1308.18311	-20.9897995
1312.90320	60.8899002
1351.24133	-19.3740005
1401.57593	14.7102003
1425.60352	3.29730010
1440.73987	-1.32319999
1476.41699	-20.5139999
1497.62585	-5.77619982
1505.77295	-9.01439953
1506.60156	35.9205017
1520.66785	14.5050001
1524.43005	-3.71869993

1533.97607	-6.41979980
1574.69324	77.5037994
1628.67639	-62.7663994
1671.48206	323.055511

B

665.426880	-275.753387
718.547302	74.3836975
759.002930	-2.44729996
774.481323	-159.697403
880.841675	11.5306997
923.461121	4.78210020
981.419922	8.08370018
1033.64441	7.50120020
1043.29297	16.5629997
1071.42151	-25.9368992
1085.14075	-3.26530004
1127.56433	30.6375999
1165.03247	-14.7121000
1189.45862	-37.3115997
1203.11646	14.2750998
1291.32739	19.8631001
1341.88831	-45.9300003
1365.72314	10.2034998
1410.27100	-19.5006008
1427.62549	-4.22910023
1434.85657	14.0881004
1477.31506	19.3089008
1483.21960	-0.486499995
1504.09070	2.81419992
1506.97534	-16.0487003
1514.54346	3.71740007
1515.42969	-0.401499987
1525.67346	-6.99959993
1608.70593	71.8676987
1618.23254	163.716797

C

633.370300	-1.49790001
659.421997	-324.197693
711.856812	-57.7467003
765.079529	-7.64050007
872.374878	3.09890008
933.367126	3.32209992
972.047424	-1.94700003
1031.94116	8.07219982
1037.11292	21.7674999
1078.11377	-39.7667007
1082.26160	-8.47939968
1126.02478	23.2823009
1160.46997	42.6726990
1194.17725	5.69700003

1211.09192	-44.8442993
1285.15771	48.6521988
1319.30029	-29.2509003
1350.27625	18.4305000
1414.72913	-8.27719975
1431.87976	-5.68639994
1437.64368	23.8299007
1472.37817	10.3958998
1489.44678	-7.98780012
1508.29956	-5.15129995
1514.42786	-12.2578001
1516.78809	-1.43910003
1524.39563	8.94890022
1528.85339	-22.5739994
1596.34436	89.1757965
1602.33459	13.3149004

D

704.194275	58.3303986
740.273499	-42.3754005
780.903198	-43.2142982
875.946716	2.77130008
932.848877	0.369100004
979.224121	-2.33080006
1036.34546	1.48360002
1050.76074	13.3255997
1072.14099	-28.0447998
1083.06116	-9.97630024
1128.03149	19.7830009
1150.62305	-4.77269983
1202.61499	-77.4170990
1203.64136	34.0685005
1307.49329	76.7975006
1320.51233	-39.7554016
1349.59985	-24.2306004
1407.05359	5.71740007
1427.41455	0.418199986
1437.13647	4.29489994
1475.50830	19.1812000
1496.14624	4.80280018
1508.24829	-13.3263998
1509.13745	10.6112003
1517.29810	3.05209994
1521.03345	8.86120033
1524.71082	-18.7765999
1592.68274	80.3644028
1627.31445	-57.1487007
1695.20215	-130.440903

- beta conformation

The following structures are also depicted in Figure S9.

A

6	0.31732	-3.70352	-0.63946
6	0.68949	-2.25688	-0.82328
7	-0.29552	-1.33627	-0.91996
6	0.09764	0.06643	-1.03796
6	-0.41052	0.88622	0.15663
7	0.57848	1.59322	0.72089
6	0.42795	2.29544	1.99116
8	1.87361	-1.84913	-0.74372
6	-0.43777	0.6695	-2.35251
8	-1.57567	0.7863	0.56836
8	4.24641	-1.07472	-0.60188
8	3.45326	1.65423	0.23044
8	-4.20809	1.66244	1.48365
8	-4.03259	1.65201	-1.3697
8	-3.58726	-1.01692	-0.68808
8	2.6629	-2.44185	2.06933
8	2.73522	4.55241	-0.35358
8	-2.5645	-2.68467	1.66638
1	-0.7534	-3.88277	-0.76476
1	0.60939	-3.9984	0.37447
1	0.88379	-4.32217	-1.3429
1	-1.27821	-1.59574	-0.97221
1	1.18195	0.07384	-1.05323
1	-0.09072	1.70313	-2.4613
1	-0.07353	0.08875	-3.20722
1	-1.53214	0.68138	-2.36624
1	1.52868	1.52038	0.3605
1	1.0437	3.19888	1.97954
1	0.73101	1.653	2.82778
1	-0.61652	2.58126	2.12685
1	3.62711	-2.45428	2.16305
1	2.51853	-2.20846	1.1331
1	4.93452	-1.24078	-1.262
1	3.41992	-1.49684	-0.91372
1	-3.91675	-1.66863	-0.04603
1	-3.02184	-0.42117	-0.14762
1	-2.15918	-1.86899	2.00473
1	-2.83596	-3.18169	2.45295
1	-3.24593	1.53536	1.54114
1	-4.32795	1.86449	0.53528
1	-4.6757	2.02618	-1.99032
1	-4.1881	0.68411	-1.36548
1	3.77903	0.78603	-0.11365
1	3.82005	1.71867	1.12614
1	3.43468	5.11773	-0.71206
1	3.09188	3.64432	-0.37024

B

6	1.57802	-2.69837	0.03979
6	1.59322	-1.19715	-0.1569
7	0.68843	-0.64333	-0.98924
6	0.54983	0.79008	-1.23159
6	-0.86951	1.13914	-0.73914
7	-0.99294	2.1646	0.12242
6	-2.24818	2.45798	0.8187
8	2.45638	-0.49202	0.41825
6	0.73724	1.12176	-2.71746
8	-1.81716	0.3934	-1.02155
8	-3.90728	-0.54826	-2.17232
8	3.95863	-1.32423	2.4713
8	4.58163	-0.02763	-1.20603
8	-1.30885	-2.85347	-1.81305
8	1.5063	4.04036	-0.21157
8	-0.78796	1.1287	3.69331
8	-1.62217	-2.07779	1.63183
8	-3.78296	-0.85196	1.91171
1	1.4628	-3.22825	-0.90922
1	0.74636	-2.9811	0.69323
1	2.51018	-2.99424	0.52353
1	-0.03835	-1.2443	-1.38722
1	1.30733	1.29251	-0.62774
1	0.63392	2.20026	-2.88705
1	1.73706	0.81891	-3.04601
1	-0.01319	0.60755	-3.32605
1	-0.16603	2.7068	0.35643
1	-2.01041	3.01889	1.72449
1	-2.74355	1.52158	1.088
1	-2.92153	3.0449	0.18427
1	4.76246	-1.38653	1.93272
1	3.29672	-0.98689	1.83524
1	5.10376	0.60368	-0.68805
1	3.77075	-0.15723	-0.67786
1	-0.89954	-3.37352	-2.52273
1	-2.24467	-2.77282	-2.06833
1	-1.42528	-1.58948	0.81358
1	-1.42409	-3.00696	1.43575
1	-4.48583	-0.84332	-1.45271
1	-3.10191	-0.23847	-1.72761
1	-3.06305	-1.50605	1.97621
1	-3.74497	-0.33711	2.73046
1	1.99132	4.85325	-0.00162
1	1.26294	4.12063	-1.14739
1	-0.09996	1.17162	3.012
1	-0.32758	1.25943	4.53592

C

6	1.54295	-2.24384	1.97025
6	1.50193	-1.14116	0.92543

7	0.31774	-1.01972	0.28956
6	-0.03823	-0.02869	-0.71266
6	-1.18983	0.81346	-0.14932
7	-1.03958	2.14499	-0.17945
6	-1.94094	3.06831	0.49733
8	2.53615	-0.51201	0.64686
6	-0.44333	-0.68859	-2.04226
8	-2.18546	0.25429	0.35846
8	4.02893	0.97462	-1.18575
8	1.96604	2.92337	-0.99428
8	-3.96056	0.0919	2.61896
8	1.52688	3.99936	1.21917
8	-3.91415	0.64232	-2.08418
8	-2.18105	-2.97319	0.72985
8	-2.00299	-3.6687	-1.73061
8	5.51047	-2.56402	0.22729
1	1.06656	-3.15524	1.59106
1	1.00641	-1.95157	2.88377
1	2.58565	-2.45263	2.21877
1	-0.43107	-1.61728	0.62744
1	0.83712	0.60344	-0.88487
1	-0.62624	0.07332	-2.81051
1	0.37009	-1.34373	-2.37168
1	-1.35371	-1.28582	-1.93295
1	-0.16933	2.5155	-0.54277
1	-1.41173	3.99864	0.72263
1	-2.29527	2.61697	1.42907
1	-2.79947	3.29897	-0.1464
1	3.43962	0.49916	-0.55364
1	4.89511	0.90986	-0.75515
1	6.16909	-2.68447	-0.47234
1	4.70095	-2.2801	-0.22505
1	-2.86473	-2.28675	0.81817
1	-2.54088	-3.7844	1.12143
1	-1.99066	-3.40334	-0.79626
1	-2.82232	-4.16714	-1.85737
1	-4.69727	0.55519	2.19413
1	-3.28658	0.04462	1.91412
1	-3.58779	0.69645	-1.1688
1	-4.86958	0.79089	-2.04403
1	2.71384	2.33462	-0.75463
1	2.15389	3.11556	-1.92691
1	1.93601	4.87404	1.203
1	1.73595	3.58799	0.37028

D

6	0.33926	-3.59562	0.78561
6	0.71254	-2.22312	0.28649
7	-0.27612	-1.38135	-0.08109
6	0.09786	-0.03508	-0.50591
6	-0.54245	0.94964	0.48676
7	0.27208	1.85533	1.03264

6	-0.11577	2.72387	2.13609
8	1.9041	-1.84571	0.17429
6	-0.37048	0.23947	-1.94376
8	-1.74495	0.80376	0.81268
8	-2.99035	0.24249	3.10547
8	4.06407	-2.45948	-1.38519
8	3.70965	-0.60383	1.94595
8	3.08285	1.78098	-0.09928
8	2.41655	3.10719	-2.35082
8	-3.406	-2.41518	-0.77511
8	-5.07017	-0.81838	-1.73987
8	-1.84604	3.0801	-1.13874
1	-0.68314	-3.8737	0.51385
1	0.42253	-3.60952	1.87913
1	1.04696	-4.32837	0.38903
1	-1.25866	-1.6462	-0.04937
1	1.18028	0.02573	-0.45492
1	-0.13682	1.26821	-2.23417
1	0.12967	-0.45238	-2.63014
1	-1.45259	0.09827	-2.03452
1	1.22672	1.91759	0.67542
1	0.7807	2.98034	2.70566
1	-0.81905	2.20062	2.78758
1	-0.58218	3.64728	1.77101
1	3.8726	-3.08288	-2.09965
1	3.22346	-2.33064	-0.9038
1	4.5578	-1.04868	1.79344
1	3.05744	-1.1547	1.46373
1	-4.18261	-2.07289	-0.30302
1	-3.78699	-2.97153	-1.47123
1	-2.55981	0.49162	2.26355
1	-3.8017	0.76712	3.15903
1	-2.16205	2.42697	-0.48744
1	-2.29931	3.90729	-0.9175
1	-4.12413	-0.60649	-1.77933
1	-5.54272	-0.00628	-1.97893
1	3.44417	1.06112	0.466
1	3.63028	2.56286	0.07437
1	2.63506	2.30614	-1.84136
1	2.52887	2.87472	-3.28394

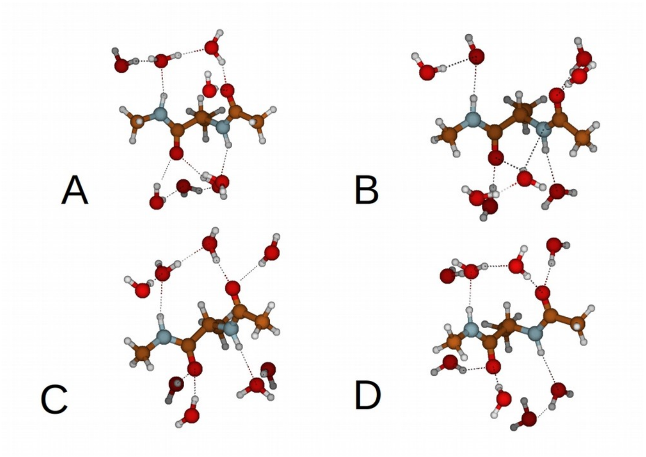

Figure S9. Pictorial representation of the representative conformations of $ADP(H_2O)_8$ free energy basins for the MD-constrained simulation of ADP in the β conformation.

Below we report the VCD signals of the structures previously reported calculated at the same level of theory. Frequencies are reported in cm^{-1}, Rotational strengths are reported in 10^{-44} esu^2cm^2

A

722.612671	69.4009018
738.783386	-27.4412003
777.765991	1.30250001
881.023071	11.5914001
929.114197	4.49739981
991.120483	-4.60750008
1037.22485	-5.42810011
1048.00122	-1.11170006
1075.20618	-9.75599957
1078.08154	16.1982002
1119.39087	16.9097996
1151.93372	57.2170982
1184.82324	19.9204998
1196.60864	-99.8728027
1299.50769	55.9385986
1310.39404	-64.0419006
1357.23987	-31.7826004
1424.63745	-12.4649000
1431.87085	-15.5176001
1442.13196	-0.338900000
1466.25842	7.93450022
1490.42358	-23.5289993
1504.05469	22.4923992
1514.83752	4.22720003
1516.83740	-16.4326992
1522.02881	32.4257011
1523.17273	-14.8015003
1570.53540	95.7016983
1628.82617	-210.114807

1654.92969	35.1435013

B

703.387695	-60.2951012
771.753113	-4.09240007
826.443726	-21.9265003
884.360779	13.3248997
917.214600	7.24630022
979.965698	-15.0219002
1031.72424	7.63320017
1037.07593	10.7697001
1072.13086	6.17770004
1091.61963	-6.65889978
1118.40417	9.78450012
1162.34583	-19.2178993
1175.88782	-58.3779984
1203.83704	20.0249004
1279.35498	54.1780014
1336.14966	-58.8218002
1365.66174	31.6299000
1396.39978	-4.66309977
1424.23889	-2.59570003
1431.41577	9.55210018
1479.10742	-20.3868999
1480.11377	-11.2627001
1500.04834	11.2795000
1503.52185	12.1812000
1512.52844	-7.59959984
1519.07275	11.2258997
1533.55457	-11.0724001
1602.01294	-325.615295
1606.96838	251.283295
1733.25281	-217.578598

C

667.271118	18.0790997
693.318420	-84.1802979
711.104675	29.7117004
771.862122	-15.9899998
880.596130	2.97420001
914.877502	4.82350016
979.285217	-3.74559999
1032.80139	15.5819998
1053.01318	12.7707005
1072.80481	-6.90170002
1096.01367	-5.96400023
1119.88391	0.587400019
1165.75842	-9.78090000
1186.54944	-54.3664017
1203.63623	9.53330040
1286.71887	38.3428001
1330.65845	-59.8772011

1360.40784	6.25969982
1403.90381	6.06500006
1429.29187	-8.10149956
1441.78235	5.67479992
1471.79346	-2.06049991
1494.74841	-10.1724005
1507.93079	-20.6394997
1509.32617	33.8572006
1512.01074	-0.830100000
1522.83826	-3.33579993
1529.60291	-9.49989986
1603.88989	-195.471603
1609.10693	294.467590

D

714.784729	-47.5946999
769.745911	2.92840004
823.988220	-62.7840996
880.989929	-21.3472004
926.688904	3.46420002
984.184204	-4.36149979
1034.86475	4.36299992
1052.46375	-2.26909995
1074.88684	3.40910006
1088.47327	2.32839990
1119.45056	22.4874001
1152.80811	-25.8787994
1181.71582	-74.2988968
1196.08862	13.2187004
1297.18872	54.0606995
1320.30798	-88.1332016
1365.98938	26.8257999
1408.45483	6.85650015
1430.03003	-18.7353992
1433.48633	4.77139997
1468.26135	-16.9104996
1491.30920	-24.6576996
1505.06763	15.5150995
1506.48218	-8.50629997
1514.11218	4.88339996
1516.50464	19.5412006
1530.91150	-3.68149996
1576.07092	51.9929008
1642.27515	-322.287415
1650.36646	139.339493

- α_R *conformation*

The following structures are also depicted in Figure S10.

A

6	-2.60314	1.95056	1.27426
6	-1.39094	1.04279	1.33751
7	-1.38533	-0.01453	0.47084
6	-0.25009	-0.92409	0.35026
6	0.8513	-0.47786	-0.62105
7	0.50966	0.30877	-1.63922
6	1.37997	0.73142	-2.73337
8	-0.43708	1.30334	2.07365
6	-0.72925	-2.33493	-0.04114
8	2.01566	-0.90339	-0.40817
8	2.55867	-3.40555	0.38923
8	1.8984	0.07229	3.22314
8	4.33462	-1.42836	-1.18072
8	3.04691	2.01855	0.21104
8	1.10196	3.73559	0.38109
8	-2.48875	1.56168	-2.72363
8	-4.08065	-1.03671	-1.45361
8	-4.39421	-2.17585	1.4909
1	-2.27158	2.92455	0.89888
1	-2.99643	2.10231	2.2844
1	-3.39984	1.57056	0.62757
1	-2.21019	-0.19056	-0.09313
1	0.24068	-0.97113	1.32532
1	-1.51035	-2.66675	0.65045
1	-1.13446	-2.34551	-1.06162
1	0.10301	-3.04328	0.00205
1	-0.44861	0.65225	-1.66251
1	2.37336	0.30466	-2.5967
1	1.45563	1.82265	-2.74571
1	0.96975	0.37936	-3.6848
1	2.52213	0.66708	2.77716
1	1.03085	0.4528	2.98684
1	0.706	3.53106	1.24127
1	1.88892	3.16435	0.31625
1	-4.98489	-0.90414	-1.78167
1	-3.70909	-1.72343	-2.03166
1	-4.29409	-1.77997	0.60702
1	-3.94086	-1.57622	2.1018
1	2.35763	-2.45786	0.26681
1	2.72972	-3.51835	1.33678
1	4.35675	-2.18354	-0.57618
1	3.41725	-1.1286	-1.14204
1	-3.42209	1.35956	-2.56207
1	-2.43059	2.52429	-2.82511
1	2.72941	1.12111	-0.00049
1	4.01024	1.9929	0.09757

B

6	-2.80288	1.2027	0.69954
6	-1.93263	0.14679	0.04387
7	-0.8015	0.60653	-0.57126
6	0.17461	-0.28989	-1.1644
6	1.22713	-0.80094	-0.15081
7	1.50328	-0.02086	0.8982
6	2.5629	-0.30979	1.85923
8	-2.22388	-1.05207	0.06553
6	0.87696	0.37345	-2.36007
8	1.7833	-1.91346	-0.32269
8	3.6247	-1.93773	-1.9386
8	-0.16628	-4.0737	0.00869
8	-1.87729	-3.3152	2.15532
8	-4.26544	-1.19414	-2.15489
8	-0.03289	3.53528	0.56323
8	-1.56589	4.56167	-1.45947
8	4.39621	2.07027	-0.13836
8	-0.40979	1.48327	3.53086
1	-2.65207	1.18596	1.784
1	-3.85299	0.95993	0.50813
1	-2.58694	2.21162	0.33731
1	-0.56798	1.59354	-0.46894
1	-0.36204	-1.18379	-1.48947
1	0.13291	0.65342	-3.11235
1	1.4362	1.2595	-2.04319
1	1.58887	-0.3288	-2.80481
1	0.96904	0.83177	1.03256
1	2.70927	-1.39072	1.90381
1	2.26367	0.05372	2.84527
1	3.49274	0.16711	1.53506
1	-3.43666	-1.31912	-1.65523
1	-4.90285	-1.01526	-1.44608
1	-1.23785	-3.43607	1.42287
1	-2.73793	-3.42925	1.72706
1	-0.40346	3.80941	1.41514
1	-0.46951	4.07534	-0.12392
1	-2.1454	5.33592	-1.39069
1	-1.92514	4.01675	-2.17733
1	3.38256	-2.73856	-2.42863
1	2.88739	-1.88821	-1.31091
1	-0.7564	-3.89334	-0.73933
1	0.59932	-3.47551	-0.11978
1	-0.73759	0.56913	3.57196
1	0.12903	1.58888	4.33006
1	5.36387	2.05406	-0.18117
1	4.16883	2.38551	0.74901

C

6	2.45454	1.75775	-0.67298
6	1.08505	1.21721	-1.01946

7	0.92377	-0.11537	-0.87159
6	-0.34808	-0.82274	-1.06924
6	-1.20826	-0.82714	0.19873
7	-0.4898	-0.74575	1.3396
6	-1.07828	-0.86533	2.67019
8	0.1416	1.96231	-1.34896
6	-0.09993	-2.26067	-1.54883
8	-2.43006	-0.97357	0.1334
8	-4.58737	-0.74547	-1.65805
8	-2.68912	1.95348	-1.36694
8	1.34569	2.07296	2.19472
8	-5.11084	-1.56179	1.3639
8	0.58806	4.36611	0.03286
8	2.56262	-0.62231	2.33883
8	3.64255	-2.08407	0.53626
8	4.73116	-2.21292	-2.1315
1	2.36932	2.43475	0.18557
1	2.82531	2.35796	-1.50937
1	3.17755	0.96668	-0.45283
1	1.69298	-0.64691	-0.47501
1	-0.9159	-0.27631	-1.82493
1	0.46632	-2.24925	-2.48581
1	0.46554	-2.83759	-0.80692
1	-1.05516	-2.76409	-1.72146
1	0.46954	-0.44155	1.2587
1	-2.03216	-0.33351	2.71488
1	-0.39227	-0.43045	3.40285
1	-1.26052	-1.91519	2.92582
1	-2.98739	2.45391	-0.59484
1	-1.71588	2.01592	-1.37754
1	0.33137	5.20955	-0.36555
1	0.40771	3.6789	-0.64528
1	3.27096	-1.47374	1.19776
1	4.57616	-2.20245	0.76119
1	4.63354	-3.15315	-1.91561
1	4.16728	-1.78383	-1.4641
1	-5.48664	-1.49896	0.46809
1	-4.16926	-1.39061	1.18741
1	-4.77877	-0.0215	-2.27121
1	-3.73611	-0.49701	-1.25249
1	1.12577	2.75195	1.52972
1	0.87094	2.34633	2.9946
1	2.40389	0.34282	2.304
1	1.85348	-1.04721	2.84959

D

6	-2.21955	-1.57134	1.67727
6	-0.82862	-1.43887	1.10012
7	-0.67619	-1.41924	-0.25318
6	0.6321	-1.20292	-0.86901
6	1.04234	0.2688	-1.07239
7	0.09177	1.20232	-1.22691

6	0.42605	2.58431	-1.5558
8	0.15779	-1.35486	1.84361
6	0.73528	-1.95285	-2.2065
8	2.23934	0.56101	-1.15091
8	1.25766	1.1076	2.26396
8	4.05404	-1.47818	-0.93649
8	-1.2146	2.41	2.50281
8	4.11375	2.60434	-0.66484
8	-2.93316	-0.40751	-1.63504
8	-5.39807	-1.24937	-0.9065
8	-3.2339	2.58221	0.44589
8	2.85476	-2.32665	2.19646
1	-2.44714	-0.66123	2.24351
1	-2.21897	-2.40114	2.3896
1	-3.00115	-1.73496	0.93103
1	-1.5087	-1.38756	-0.81712
1	1.37108	-1.60989	-0.17906
1	0.36555	-2.97703	-2.08917
1	0.16373	-1.4584	-3.00443
1	1.78478	-1.99775	-2.5057
1	-0.87056	0.93311	-1.07952
1	1.21565	2.94292	-0.88874
1	-0.46268	3.204	-1.41475
1	0.77126	2.67314	-2.59219
1	0.92328	0.20686	2.38892
1	0.5278	1.69947	2.51197
1	2.76386	-3.28138	2.33099
1	1.9442	-1.99545	2.07859
1	-3.24027	0.47896	-1.89083
1	-2.76312	-0.87721	-2.4715
1	-4.47826	-0.99144	-1.08362
1	-5.7187	-1.72092	-1.68815
1	3.48378	-0.68632	-0.90379
1	4.13265	-1.78919	-0.02041
1	3.43016	1.93231	-0.84094
1	4.67836	2.25264	0.03821
1	-1.80049	2.27374	1.73719
1	-1.62947	3.13077	3.0004
1	-3.03199	3.13984	-0.32177
1	-4.20021	2.5091	0.38834

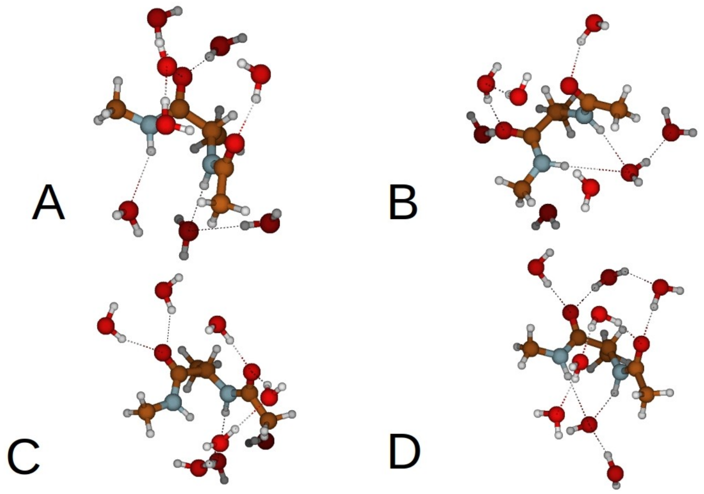

Figure S10. Pictorial representation of the representative conformations of ADP$(H_2O)_8$ free energy basins for the MD-constrained simulation of ADP in the α_R conformation.

Below we report the VCD signals of the structures previously reported calculated at the same level of theory. Frequencies are reported in cm^{-1}, Rotational strengths are reported in 10^{-44} esu^2cm^2

A

676.739502	-99.7845001
709.961304	132.212006
747.253723	257.272491
770.436096	-111.707703
867.668884	-11.0230999
931.120300	1.16030002
958.500916	-0.224199995
1014.62958	-15.1556997
1049.24353	5.07569981
1067.53711	-7.37209988
1107.53467	-8.43169975
1127.84143	18.8320007
1158.69019	0.546299994
1171.33411	-84.6862030
1199.18018	8.03839970
1291.69568	-52.4846001
1328.18433	85.5898972
1342.37122	80.8163986
1394.01208	-23.4736996
1422.62268	4.15390015
1440.68665	16.6200008
1465.28931	-13.8175001
1495.39038	-15.6890001
1509.91797	9.52050018
1514.75818	8.82830048
1518.38293	-9.01920033
1522.71497	17.9955006

1532.39624	-11.3080997
1557.28613	-29.0414009
1624.00378	-68.6856995

B

672.562317	42.4496994
732.559692	72.8705978
774.023682	-46.8646011
860.773621	-11.2292995
926.279175	8.86999965E-02
959.263123	-5.77229977
1013.78912	-20.5939007
1049.05444	-13.8207998
1069.03845	0.809400022
1111.57825	-19.9500008
1129.30945	23.8321991
1161.25464	23.2061005
1183.71289	-65.2354965
1198.41907	2.12890005
1294.81006	-90.2726974
1314.94531	105.026001
1327.34131	71.9923019
1369.24036	-25.9372997
1428.99976	4.82950020
1435.39124	12.8352003
1453.01868	14.6044998
1492.52576	-22.1564999
1503.31445	-7.41930008
1506.60339	5.65129995
1515.79749	9.08069992
1522.01282	-33.5559998
1529.59656	-1.52680004
1570.08826	-106.399803
1610.70813	44.3362999
1664.68616	-52.2991982

C

633.354919	-132.543900
674.600891	-123.407097
719.029175	-41.9910011
745.612305	9.79549980
867.985474	3.37220001
924.604187	-2.04460001
964.776001	6.31680012
1016.59137	-33.5354004
1054.84851	-34.1997986
1081.09277	17.2052994
1115.13147	1.14960003
1128.73486	13.6913996
1155.51855	-5.38339996
1173.14038	-76.5651016
1196.36804	9.09500027

1309.31323	-42.4698982
1322.93799	55.0171013
1333.77039	87.5696030
1372.30432	-21.1373997
1434.16785	10.6581001
1446.50745	34.6156006
1469.34375	16.3239002
1493.11145	-13.3255997
1501.99487	-16.6378002
1511.98853	10.9730997
1518.07141	11.6222000
1522.30383	-2.68300009
1531.67505	6.59450006
1572.05481	-386.360992
1600.89929	183.476898

D

610.302002	74.2958984
656.473877	-10.4288998
673.711914	100.163803
868.453003	-4.39870024
936.064087	-2.90499997
965.552490	2.32100010
1012.92670	-32.6871986
1041.23572	-0.745500028
1070.32812	6.93510008
1105.58264	-12.2625999
1128.12024	17.6100998
1157.65710	4.68860006
1182.32544	-84.5982971
1203.60229	12.4150000
1295.99573	-40.3694992
1318.56299	55.7606010
1327.42224	73.5095978
1393.02783	-30.1179008
1424.78784	17.1905994
1427.85889	2.00979996
1473.83350	2.98990011
1495.65076	-23.6490993
1499.84814	-22.4090004
1507.15723	9.06040001
1514.66895	5.30989981
1524.52197	-0.776700020
1529.80750	16.5009995
1553.91528	-82.4775009
1597.01794	11.7132998
1741.38806	-279.561707

S5-Analysis of the convergence.

In order to evaluate the convergence of our results we proceeded as follows. We first divided the trajectory (as emerged from the rEMCS procedure) into three portions. On each of the three portions we performed the analysis of the conformations through Essential Dynamics (see Section S.1). We then compared the associated eigenvectors of the corresponding covariance matrix and the projections on the first two eigenvectors.

1-L-ALANINE

In the Figure S9 we report the convergence analysis for 1-L-alanine with 7 water molecules. A very high degree of similarity can be observed in the three sub-sets.

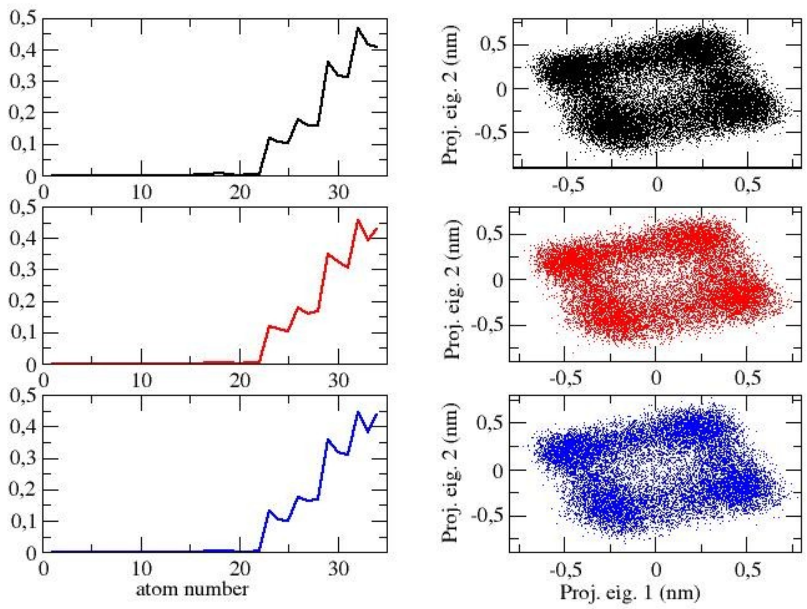

Figure S9. Composition of the first eigenvector of the covariance matrix (left panels) and the projection of the three portions of the rEMCS trajectory on the first two eigenvectors (right panels). The three spectra of the eigenvalues, basically indistinguishable from the one shown in the main text, are not reported for the sake of clarity.

Analysis of the probability of the four conformational basins resulted in the following values:
p_A^{first} = 0.32 ; p_A^{second} = 0.32; p_A^{third} = 0.31
p_B^{first} = 0.30 ; p_B^{second} = 0.29; p_B^{third} = 0.29
p_C^{first} = 0.14 ; p_C^{second} = 0.16; p_C^{third} = 0.15
p_D^{first} = 0.17 ; p_D^{second} = 0.19; p_C^{third} = 0.17

The probabilities across the three subsets vary by no more than 2%.

2- (1S,2S)-trans-1-amino-2-indanol

In the Figure S10 we report the composition of the first two eigenvectors obtained from the covariance matrix diagonalization for each of the three portions of each of the four rEMCS trajectories of 2- (1S,2S)-trans-1-amino-2-indanol and 3 dmso molecules. A very high degree of similarity can be observed in the three sub-sets.

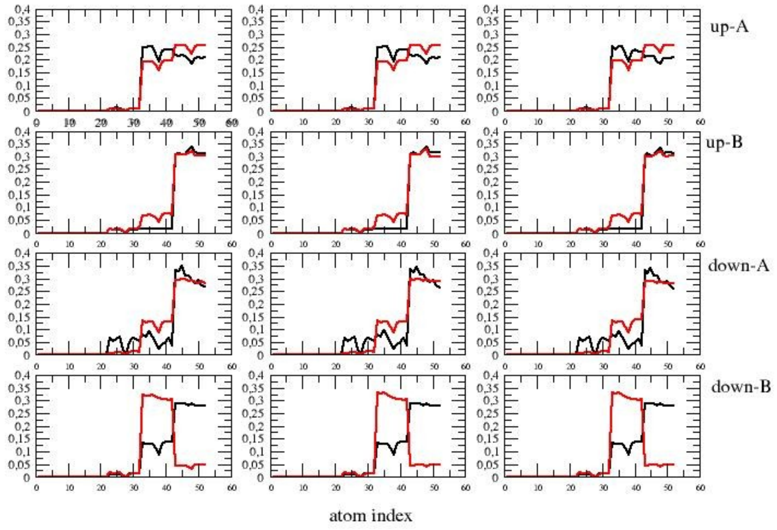

Figure S10. Atom composition of the first two eigenvectors (firs, black; second, red) of the covariance matrix for the three portions of each of the four rEMCS trajectories of the (1S,2S)-trans-1-amino-2-indanol with 3 dmso molecules. Each column corresponds to a specifici portion.

In the Figure S11 we also report the corresponding projections on the first two eigenvectors. Also in this case the high degree of resembliance is remarkable.

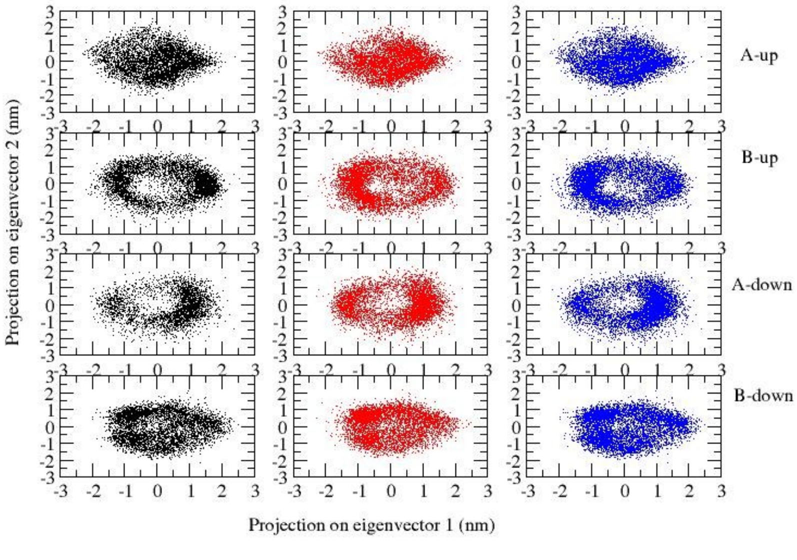

Figure S11. Projection on the first two eigenvectors for the first (black), second (red) and third (blue) portion of the rEMCS trajectory of the (1S,2S)-trans-1-amino-2-indanol with three dmso molecules in the A-up, B-up, A-down and B-down states.

Analysis of the probability of the four conformational basins resulted in the following values:

$p_{1A\text{-}up}^{first} = 0.18$; $p_{1A\text{-}up}^{second} = 0.19$; $p_{1A\text{-}up}^{third} = 0.19$

$p_{1B\text{-}up}^{first} = 0.10$; $p_{1B\text{-}up}^{second} = 0.09$; $p_{1B\text{-}up}^{third} = 0.08$

$p_{1A\text{-}down}^{first} = 0.24$; $p_{1A\text{-}down}^{second} = 0.26$; $p_{1A\text{-}down}^{third} = 0.25$

$p_{1B\text{-}down}^{first} = 0.13$; $p_{1B\text{-}down}^{second} = 0.11$; $p_{1B\text{-}down}^{third} = 0.11$

$p_{2A\text{-}up}^{first} = 0.07$; $p_{2A\text{-}up}^{second} = 0.07$; $p_{2A\text{-}up}^{third} = 0.06$
$p_{2B\text{-}up}^{first} = 0.06$; $p_{2B\text{-}up}^{second} = 0.05$; $p_{2B\text{-}up}^{third} = 0.06$
$p_{2A\text{-}down}^{first} = 0.16$; $p_{2A\text{-}down}^{second} = 0.15$; $p_{2A\text{-}down}^{third} = 0.14$
$p_{2B\text{-}down}^{first} = 0.09$; $p_{2B\text{-}down}^{second} = 0.10$; $p_{2B\text{-}down}^{third} = 0.10$

Note that the superscripts first, second and third refer to the three portions of the corresponding trajectory. The probabilities across the three subsets vary by no more than 2%.

3- CAPPED DI-ALANINE

In the Figure S12 we report the projections on the first two eigenvectors obtained from the covariance matrix diagonalization for the three portions of each of the three rEMCS trajectories of aqueous capped alanine dipeptide (ADP) with eight water molecules.

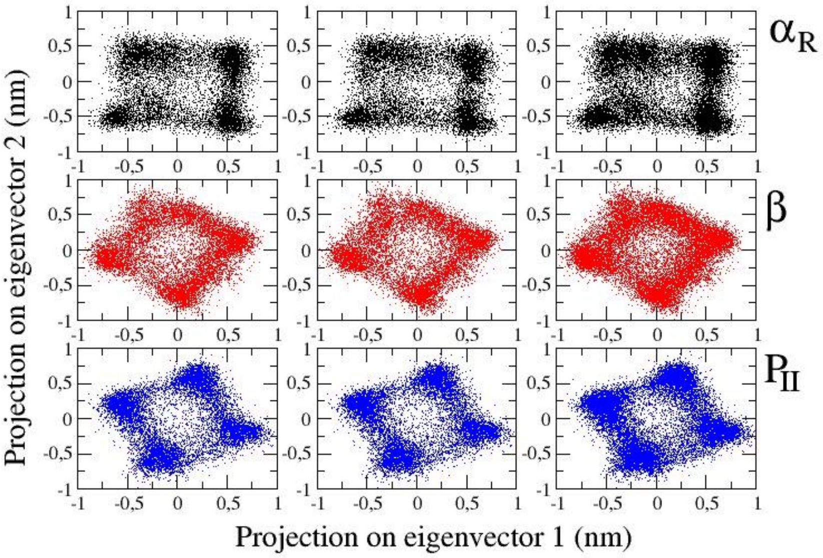

Figure S12. Projection on the first two eigenvectors for the first (black), second (red) and third (blue) portion of each of the rEMCS trajectories of the ADP with eight water molecules.

Analysis of the probability of the three conformational basins in the trree portions of the trajectory resulted in the following values:

$p_A^{\alpha\text{-first}} = 0.32$; $p_A^{\alpha\text{-second}} = 0.32$; $p_A^{\alpha\text{-third}} = 0.31$
$p_B^{\alpha\text{-first}} = 0.30$; $p_B^{\alpha\text{-second}} = 0.29$; $p_B^{\alpha\text{--third}} = 0.29$
$p_C^{\alpha\text{-first}} = 0.14$; $p_C^{\alpha\text{-second}} = 0.16$; $p_C^{\alpha\text{--third}} = 0.15$
$p_D^{\alpha\text{-first}} = 0.17$; $p_D^{\alpha\text{-second}} = 0.19$; $p_D^{\alpha\text{--third}} = 0.18$
$p_A^{\beta\text{-first}} = 0.32$; $p_A^{\beta\text{-second}} = 0.32$; $p_A^{\beta\text{--third}} = 0.31$
$p_B^{\beta\text{-first}} = 0.30$; $p_B^{\beta\text{-second}} = 0.29$; $p_B^{\beta\text{--third}} = 0.29$
$p_C^{\beta\text{-first}} = 0.14$; $p_C^{\beta\text{-second}} = 0.16$; $p_C^{\beta\text{--third}} = 0.15$
$p_D^{\beta\text{-first}} = 0.17$; $p_D^{\beta\text{-second}} = 0.19$; $p_D^{\beta\text{-third}} = 0.19$
$p_A^{PII\text{-first}} = 0.32$; $p_A^{PII\text{-second}} = 0.32$; $p_A^{PII\text{-third}} = 0.31$

$p_B^{PII\text{-}first} = 0.30$; $p_B^{PII\text{-}second} = 0.29$; $p_B^{PII\text{-}third} = 0.28$
$p_C^{PII\text{-}first} = 0.14$; $p_C^{PII\text{-}second} = 0.16$; $p_C^{PII\text{-}third} = 0.17$
$p_D^{PII\text{-}first} = 0.17$; $p_D^{PII\text{-}second} = 0.19$; $p_D^{PII\text{-}third} = 0.18$

Note that the superscripts first, second and third refer to the three portions of the corresponding trajectory. The probabilities across the three subsets vary by no more than 2%.

S6 - Quantitative evaluation of the agreement between calculated and experimental spectra

In this section we quantified our results with respect to experimental data and to other computational studies. When possible, i.e. when explicit values of the experimental frequencies were avalible, we calculated the Root Mean Square Deviation (RMSD) according to

$$RMSD = \sqrt{\frac{\sum (freq_{calc} - freq_{\exp})^2}{n_{freq}}} \qquad \text{S6.1}$$

In the other cases, i.e. when experimental frequencies were not explicitly reported, we have compared the Spectrum-RMSD, i.e. Root Mean Square Deviation of the experimental points (indicated as maxima or minima in the spectra, i.e. in the freq/Intensity experimental plane) with the corresponding calculated points according to

$$Spectrum - RMSD = \sqrt{\frac{\sum (freq_{calc} - freq_{\exp})^2 + (Intensity_{calc} - Intensity_{\exp})^2}{n_{freq}}} \qquad \text{S6.2}$$

Note that all these analyses were performed after having applied a systematic shift to all the calculated spectra in order to obtain the best fit to the experimental data and after having normalized the calculated and experimental intensity.

1- L- Alainine

In the Table below we report the experimental[1] and calculated frequencies. Note that for the calculated frequencies we report the maxima of the curves after the inclusion of all the representative structures of the corresponding cluster basin (see main text for additional details)

Experimental (cm^{-1})	Calculated (cm^{-1}) (with PCM)
1418	1419 (1429)
1358	1357 (1364)
1306	1322 (1332)
1221	1210 (1221)
1139	1163 (1163)
1117	1114 (1114)

These data produced a RMSD (after the shift of the frequencies, see equation 6S.1) of 12.3 cm^{-1}.

1 Diem, M.; Photos, E.; Khouri, H.; Nafie, L. A. Vibrational Circular Dichroism in Amino Acids and Peptides. 3. Solution- and Solid-Phase Spectra of Alanine and Serine. J. Am. Chem. Soc. 1979, 101, 6829−6837.

Considering both the frequencies and intensities of the maxima (and the minima) of the spectra, reported in the Table below, and depicted in the Figure 6 of the main text, we obtained RMSD values of 10.3 and 15 (with PCM) using the equation S6.2.

Frequencies (cm^{-1}) and normalized intensity

Experimental (cm^{-1})	Calculated (cm^{-1})	Calculated with PCM (cm^{-1})
1117. -58.	1130 -10	1130 -60.
1139. -57.	1150 -50.	1159 -50.
1221. 58.	1212 22	1220 41
1306. 176.	1321 157.	1331 179
1357. -299.	1355 -292	1363 -289
1418. 56.	1420 113.	1429 139

2- (1S,2S)-trans-1-amino-2-indanol

In this case explicit experimental frequencies are not available, but the spectrum is. Therefore, we considered the maxima and minima of the experimental and calculated spectra (see Figure 12 in the main text), as reported in the table below, and applied the Spectrum-RMSD measure (equation S6.2). Note that for the calculated frequencies we report the maxima of the curves including all the representative structures of the corresponding cluster basin (see main text for additional details)

Frequencies (cm^{-1}) and normalized intensity

Experimental	Calculated
1121. -2.9	1124. -0.9
1152, 1.7	1141 0.48
1169. -0.2	1162 -0.3
1187. 1.2	1179. 0.53
1214. -2.2	1218. -0.6
1239. 0.7	1239. 0.8
1261. 0.2	1258. 1.9
1279. 0.5	1280. 0.6
1314. -1.4	1304. -0.8
1350. 4.4	1338. 1.4
1380. 7.2	1390. 7.1
1441. 2.2	1445. 2.6
1461. 3.2	1475. 4.0
1478. 0.9	1487. 3.0
1538. 0.8	1538. 0.0
1576. 0.67	1586. 1.5
1619. 2.97	1629. 1.7
1673. 0.6	1673. 1.49

In this case, from equation S6.2, we obtained a value of 8.0 comparable with the ones obtained in the case of alanine.

3- Di-alanine

Also in this case, similarly to the previous one, we directly evaluated the Spectrum-RMSD (equation S6.2) by using the results reported in the Figure 17 of the main text - and summarized in the Table below - obtaining a value of 615, i.e. significanly higher than the previous cases.

Frequencies (cm^{-1}) and normalized intensity

Experimental		Calculated	
1206.	2.9		
1217.	-2.9		
1222.	2.3	1220.	1.4
1231.	2.25	1233.	7.25
1235.	-3.6	1237.	0.6
1245.	-1.	1246.	8.0
1253.	2.7	1254.	8.1
1261	4.9	1261	4.9
1288.	-4.9	1288.	-4.9
1284.	-10.		
1291.	-8.7	1291.	-8.7
1303.	13.8	1301.	15.8
1313.	15.2	1317.	19.2
1326.	3.6	1325.	-2.0
1331.	-3.7	1331.	-1.7
1336.	2.7	1337.	-2.7
1346.	8.5		
1355.	-1.1	1357.	-1.1
1366.	5.4	1368.	1.4
1379.	9.9	1369.	3.9
1389.	-0.2		
1396.	1.8	1400.	1.0

The limited agreement with the experimental data is certainly to be ascribed to the inability of our model to reproduce the fine-structure characterizing (in particular) the red side of the experimental spectrum. In fact, if we restrict the Spectrum-RMSD to the central part of the spectrum (namely 1260-1350 cm^{-1}), we get a value of 9.6, in line with the correpsonding values fot the other two systems.
